# TIGIT^+^ iTregs elicited by human regulatory macrophages control T cell immunity

**DOI:** 10.1038/s41467-018-05167-8

**Published:** 2018-07-20

**Authors:** Paloma Riquelme, Jan Haarer, Anja Kammler, Lisa Walter, Stefan Tomiuk, Norbert Ahrens, Anja K. Wege, Ivan Goecze, Daniel Zecher, Bernhard Banas, Rainer Spang, Fred Fändrich, Manfred B. Lutz, Birgit Sawitzki, Hans J. Schlitt, Jordi Ochando, Edward K. Geissler, James A. Hutchinson

**Affiliations:** 1 Department of Surgery, University Hospital Regensburg, Franz-Josef-Strauss-Allee 11, Regensburg, 93053 Germany; 2 Miltenyi Biotec GmbH, Friedrich-Ebert-Str. 68, 51429 Bergisch Gladbach, Germany; 3 Transfusion Medicine, Institute for Clinical Chemistry, University Hospital Regensburg, Franz-Josef-Strauss-Allee 11, 93053 Regensburg, Germany; 4 Department of Gynecology and Obstetrics, University Medical Center Regensburg, Landshuter Straße 65, 93053 Regensburg, Germany; 5 Department of Nephrology, University Hospital Regensburg, Franz-Josef-Strauss-Allee 11, 93053 Regensburg, Germany; 6 Department of Statistical Bioinformatics, Institute for Functional Genomics, University of Regensburg, Am BioPark 9, 93053 Regensburg, Germany; 7 Department of Surgery, University Hospital Schleswig-Holstein, Arnold-Heller-Straße 3, 24015 Kiel, Germany; 8 Institute for Virology and Immunobiology, University of Würzburg, Versbacher Str. 7, 97078 Würzburg, Germany; 9 Institute for Medical Immunology, Berlin Charité University Hospital, Augustenburger Platz 1, 13353 Berlin, Germany; 10 Inmunología de Trasplantes, Centro Nacional de Microbiología, Cta. Majadahonda-Pozuelo Km 2, 28220 Madrid, Spain

## Abstract

Human regulatory macrophages (Mreg) have shown early clinical promise as a cell-based adjunct immunosuppressive therapy in solid organ transplantation. It is hypothesised that recipient CD4^+^ T cell responses are actively regulated through direct allorecognition of donor-derived Mregs. Here we show that human Mregs convert allogeneic CD4^+^ T cells to IL-10-producing, TIGIT^+^ FoxP3^+^-induced regulatory T cells that non-specifically suppress bystander T cells and inhibit dendritic cell maturation. Differentiation of Mreg-induced Tregs relies on multiple non-redundant mechanisms that are not exclusive to interaction of Mregs and T cells, including signals mediated by indoleamine 2,3-dioxygenase, TGF-β, retinoic acid, Notch and progestagen-associated endometrial protein. Preoperative administration of donor-derived Mregs to living-donor kidney transplant recipients results in an acute increase in circulating TIGIT^+^ Tregs. These results suggest a feed-forward mechanism by which Mreg treatment promotes allograft acceptance through rapid induction of direct-pathway Tregs.

## Abstract (web summary)

Regulatory macrophages (Mreg) can directly suppress T effector cell responses. Here the authors show that human Mreg also elicit TIGIT^+^ regulatory T cells by integrating multiple differentiation signals, and that donor Mreg-induced recipient Tregs may promote kidney transplant acceptance in patients.

## Introduction

Adoptive transfer of immunoregulatory cells as a means of establishing transplantation tolerance is a common experimental technique, but its clinical application is only now receiving serious attention^1^. Several classes of immunoregulatory cells are currently being developed as adjunct immunosuppressive agents for use in solid organ transplantation, including different sorts of regulatory T cells (Treg)^2^ and suppressive myeloid cells^3^. The human regulatory macrophage (Mreg) is a promising candidate cell type that is already far advanced in its development as a cell-based medicinal product^4^. Mregs are currently being investigated in the *ONEmreg12* trial, a phase-I/II study of Mreg therapy as a means of safely minimising maintenance immunosuppression in kidney transplant recipients (clinicaltrials.gov: NCT02085629).

Human Mregs reflect a unique state of macrophage differentiation, distinguished from macrophages in other activation states by their mode of derivation, cell-surface phenotype, stable DHRS9 expression^5^ and potent suppressor function. Human Mregs develop from macrophage colony-stimulating factor (M-CSF; CSF1)-stimulated CD14^+^ peripheral blood monocytes^6^ cultured with high concentrations of human serum^7^ through a time-dependent process^8^ that requires contact with a plastic surface, ligation of FcγRIII (CD16) by serum immunoglobulin^9^ and exposure to other serum factors. Human Mregs prevent mitogen-stimulated allogeneic T cell proliferation in vitro through interferon (IFN)-γ-induced indoleamine 2,3-dioxygenase (IDO) activity, as well as mediating a contact-dependent deletion of activated allogeneic T cells^10^. From a technological perspective, the simplicity of Mreg production and their stable suppressive activity are qualities that lend themselves well to a cell-based therapeutic product for use as an immunosuppressive, anti-inflammatory or tissue-reparative therapy^11^.

Preclinical experiments in a heterotopic mouse heart transplant model demonstrated that intravenous (i.v.) injection of donor-strain Mregs conferred long-term transplant acceptance to non-immunosuppressed, fully allogeneic recipients^12^. Importantly, this allograft-protective effect was not simply due to alloantigen exposure but depended upon Mregs expressing inducible nitric oxide synthase. Following i.v. injection into mice, allogeneic Mregs rapidly accumulated in lung, liver and spleen, but not in lymph nodes, where a proportion of transferred Mregs persisted for up to 4 weeks. Importantly, these experiments showed that living Mregs exerted a graft-protective effect that endured beyond their lifespan, which was likely mediated by recipient T cells. Although mouse and human Mregs suppress allogeneic T cell proliferation and effector functions in vitro, it remains controversial whether they can control recipient alloimmune responses in vivo through direct interaction of transferred cells with recipient T cells.

Here we explore the possibility that adoptive transfer of donor-derived Mregs to an allogeneic recipient prior to solid organ transplantation induces recipient Tregs through direct interactions between donor and recipient cells. It is hypothesised that transient Mreg-induced Treg responses might engender more stable regulatory responses through a feed-forward mechanism sustained by recipient antigen-presenting cells and Tregs. This study investigates phenotypic and functional changes wrought by human Mregs upon allogeneic CD4^+^ T cells in coculture in order to identify markers that might allow us to infer direct interactions between donor-derived Mregs and recipient T cells in Mreg-treated patients. In coculture and humanised mouse experiments, a fraction of CD4^+^ T cells converts to interleukin (IL)-10-expressing-induced Tregs (iTregs) through an activation-dependent process reliant upon CD28, IDO, retinoic acid, transforming growth factor-β (TGF-β), canonical Notch signalling and progestagen-associated endometrial protein (PAEP). After i.v. administration of donor-derived Mregs, increased TIGIT^+^ Treg frequencies are observed in the peripheral blood of prospective kidney transplant recipients. Taken together, these findings suggest TIGIT^+^ iTreg elicited by allogeneic Mreg therapy could play a mechanistically important role in its tolerogenic effects.

## Results

### Steady-state characteristics of human Mregs

**Fig. 1:**
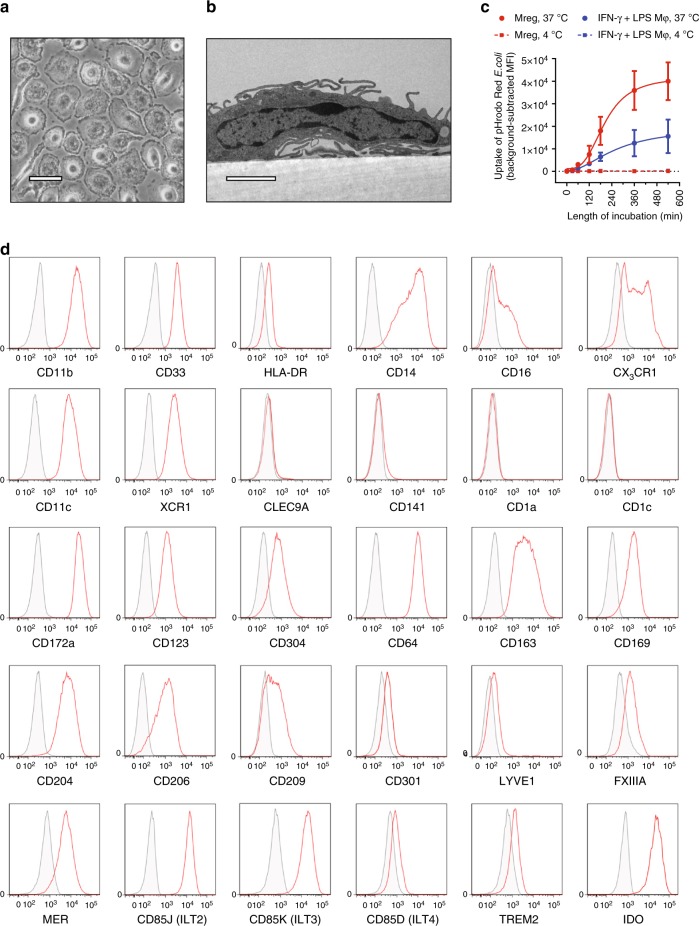
Steady-state characteristics of human Mregs. **a** Plastic-adherent Mregs acquired a distinctive morphology (bar = 50 μm). **b** Transmission electron micrographs of Mregs revealed typical morphological features of activated human macrophages (bar = 2 μm). **c** Phagocytic uptake of fluorescently labelled, fixed *E. coli* by human Mregs and classically (IFN-γ + LPS) activated macrophages over a 9-h time course (mean ± sd; *n* = 3). **d** Phenotyping of human Mregs by flow cytometry confirmed that Mregs are monocyte-derived macrophages: red = specific signal; grey = isotype control (representative of *n* = 6)

Human Mregs arose from purified CD14^+^ peripheral blood monocytes cultured in medium supplemented with human AB serum and M-CSF (CSF1) for 6 days prior to stimulation with IFN-γ for 18–24 h. Mregs adopted a distinctive tessellating, epithelioid morphology to form almost confluent monolayers (Fig. 1a). Individual cells were large and densely granular with a prominent central body and thin cytoplasmic skirt that spread symmetrically over the surface of the culture vessel. Ultrastructural examination confirmed the impression of flattened cells adhering very closely to the underlying plastic surface (Fig. 1b). Membranous processes extended from the perimeter and upper surface of Mregs, and their nuclei appeared active with abundant fine chromatin—features typical of activated human macrophages. Over a 9-h time course, Mregs exhibited a higher rate and absolute capacity for phagocytosis of fixed, fluorescently labelled *Escherichia coli* than classically (IFN-γ + lipopolysaccharide; LPS) activated macrophages (Fig. 1c).

Human Mregs expressed generic markers of human macrophages, including CD11b, CD33 and human leukocyte antigen (HLA)-DR (Fig. 1d). Unlike other monocyte-derived macrophages, Mregs expressed relatively low levels of CD14 and cell-surface CD16. Similar to CD14^low^ non-classical monocytes, Mregs expressed CX_3_CR1. Mregs uniformly expressed CD11c and XCR1, but they lacked CLEC9a and CD141 expression, so Mregs are not cDC1-like cells. Mregs expressed CD172a (SIRPα) but not CD1a or CD1c; therefore, Mregs are not cDC2-like cells. In common with pDC, human Mregs expressed CD123 (IL-3R) and CD304 (NRP1). Mregs expressed typical markers of tissue-resident macrophage subsets, including CD64, CD163, CD169, CD204 (MSR), CD206 (MMR), CD209, CD301 (MGL1), LYVE-1, Factor XIIIa and MER. A variety of inhibitory receptors were expressed by Mregs, including members of the LILR (ILT) family and TREM2. Intracellular staining for IDO revealed homogeneous expression by Mregs. A survey of extra- and intracellular expression of costimulatory and co-inhibitory ligands revealed human Mregs were CD80^−/low^ CD86^+^ CD273^+^ CD274^+^ CD275^−^ CD279^+^ CD270^+^ CD112^+^ CD155^+^ Galectin-9^+^ CD48^+^ CD252^+^ CD70^−^ GITRL^+^ CD153^+^ CD258^+^ CD160^−^ CD178^−^ CD40^+^ CD253^−^ (Supplementary Figure 1). Given their mode of derivation, morphology and cell-surface phenotype, human Mregs are best classified as a subset of monocyte-derived macrophages^13^.

### Mreg-mediated enrichment of IL-10-producing FoxP3^+^ T cells

**Fig. 2:**
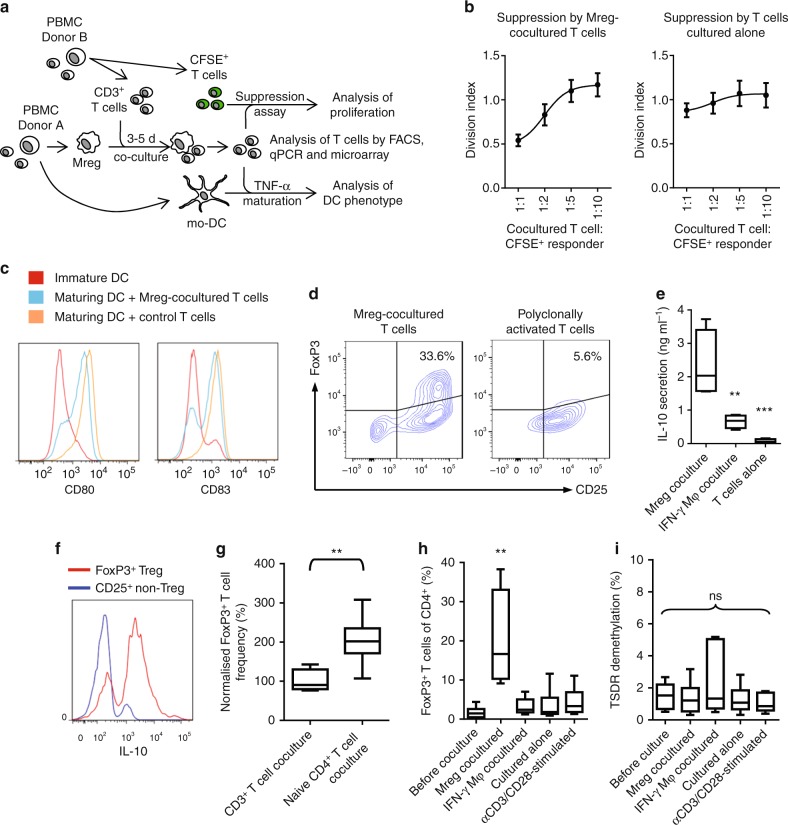
Mreg-cocultured T cells inhibit T-cell proliferation and mo-DC maturation. The functional consequences of exposing CD3^+^ T cells to allogeneic Mregs were investigated in coculture experiments. **a** Human Mregs generated from CD14^+^ monocytes were cultured together with allogeneic T cells for 5 days. T cells were then analysed by flow cytometry, qPCR, microarray and functional assays. **b** Proliferation of CFSE-labelled responder CD4^+^ T cells stimulated with plate-bound αCD3 was inhibited by allogeneic Mreg-cocultured T cells to a greater degree than by T cells cultured alone for 5 days (*n* = 6; mean ± sd). **c** CD4^+^ T cells flow-sorted from Mreg cocultures antagonised upregulation of CD80 and CD83 by mo-DCs stimulated with 50 ng ml^−1^ TNFα for 2 days (representative of *n* = 6). **d** CD3^+^ T cells cocultured with allogeneic Mregs for 5 days were enriched for FoxP3^+^ T cells that were readily discriminated from CD25^+^ FoxP3^−/low^ T cells activated by αCD3/αCD28 stimulation for 5 days (representative of *n* = 4). **e** Twenty-four-hour secretion of IL-10 by αCD3-stimulated CD4^+^ T cells re-isolated from allogeneic Mreg cocultures (*n* = 4; KW). **f** Most IL-10-producing T cells from Mreg cocultures were FoxP3^+^ T cells and vice versa (representative of *n* = 6 donors). **g** Enrichment of FoxP3^+^ T cells from naive CD25^−^ CD45RA^+^ CD4^+^ T cells, which were predominantly FoxP3^−^, was significantly more efficient than enrichment from unfractionated CD3^+^ T cells (*n* = 6; MW). **h** Coculture with allogeneic Mregs led to a significant enrichment of FoxP3^+^ T cells from a starting population of unfractionated CD3^+^ T cells. By contrast, CD3^+^ T cells that were unstimulated, polyclonally activated or cocultured with IFN-γ Mφ were not enriched for FoxP3^+^ T cells (*n* = 6; KW). **i** No increase in TSDR demethylation was observed in CD3^+^ T cells cocultured with Mregs; therefore, enrichment of FoxP3^+^ T cells reflects conversion of non-Tregs (*n* = 6; KW)

The consequences of exposing CD3^+^ T cells to allogeneic Mregs in vitro were investigated in coculture experiments (Fig. 2a). To gauge their capacity for bystander suppression, Mreg-cocultured T cells were re-isolated and mixed with autologous carboxyfluorescein succinimidyl ester (CFSE)-labelled responder T cells (Fig. 2b). At suppressor:responder ratios of >1:5, responder T cell proliferation stimulated with plate-bound anti-CD3 was inhibited by Mreg-cocultured T cells, whereas T cells cultured in the absence of Mregs were not suppressive. To test whether Mreg-cocultured T cells inhibited monocyte-derived dendritic cell (mo-DC) maturation, Mreg-cocultured allogeneic CD4^+^ T cells were flow-sorted and added at a 2:1 ratio to immature mo-DCs. These mo-DCs were then stimulated with tumour necrosis factor alpha (TNFα) over 2 days before assessing their maturation state. Mreg-cocultured T cells partially inhibited the upregulation of CD80 and CD83 (Fig. 2c). Mreg-cocultured allogeneic CD4^+^ T cells were enriched for a CD25^+^FoxP3^+^ subset (Fig. 2d and Supplementary Figure 2). An equivalent population did not emerge from T cell cultures after stimulation with plate-bound anti-CD3 for 5 days in the absence of Mregs. Twenty-four-hour secretion of IL-10 by anti-CD3-stimulated CD4^+^ T cells re-isolated from allogeneic Mreg cocultures was significantly greater than IL-10 production under control conditions (Fig. 2e). FoxP3^+^ Tregs were primarily responsible for this IL-10 secretion (Fig. 2f). Together, these findings show that Mregs enriched cocultured T cells for IL-10-producing FoxP3^+^ Tregs. This experimental system serves as the basis for subsequent in vitro experiments.

The emergence of FoxP3^+^ T cells after Mreg coculture could be explained by proliferation or passive enrichment of FoxP3^+^ Tregs present at the start of coculture, or by conversion from FoxP3^−^ T cells. To distinguish between these possibilities, Mregs were cocultured with either unfractionated CD3^+^ T cells or negatively isolated naive CD4^+^ T cells, which typically contained fewer than 2.1 ± 0.9% FoxP3^+^ T cells. Despite naive T cell preparations containing fewer FoxP3^+^ Tregs prior to Mreg coculture, generation of FoxP3^+^ T cells from naive allogeneic CD4^+^ T cells was significantly more effective than from unfractionated T cells (Fig. 2g). Enrichment of FoxP3^+^ T cells within 5 days was observed when T cells were cocultured with Mregs, but not following coculture with control IFN-γ-stimulated macrophages (IFN-γ Mφ) from the same monocyte donors (Fig. 2h and Supplementary Figure 3). No enrichment of FoxP3^+^ T cells was observed when T cells were cultured in the absence of Mregs, either with or without polyclonal stimulation using anti-CD3/CD28 beads. Hence, enrichment of FoxP3^+^ T cells appeared to be a specialised function of Mregs, as opposed to a general consequence of T cell activation. Helios was expressed by a minority (5.0 ± 0.68%; *n* = 6) of FoxP3^+^ T cells suggesting that Mreg-enriched FoxP3^+^ T cells do not principally derive from nTregs. Consistent with this interpretation, Treg-specific demethylated region (TSDR) demethylation in Mreg-cocultured T cells was only 1.35 ± 1.03% (Fig. 2i). Therefore, Mreg coculture leads to induction of FoxP3 expression in previously FoxP3^–^ CD4^+^ T cells.

**Fig. 3:**
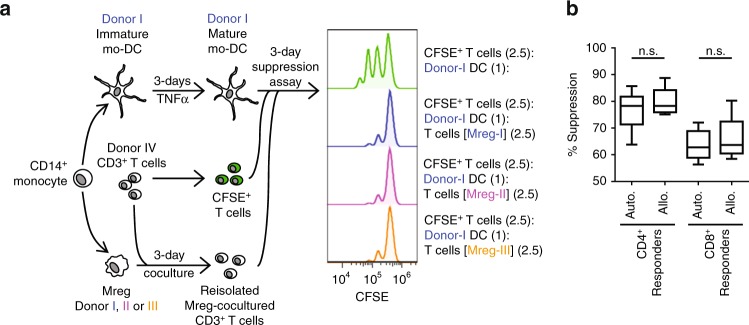
Suppression by Mreg-cocultured CD4^+^ T cells is unspecific. **a** Secondary suppression assays were performed to assess the specificity of Mreg-cocultured T cell-mediated suppression of allogeneic DC-stimulated T cell proliferation. **b** Quantification of bystander suppressor activity of Mreg-cocultured T cells over allo-stimulated CD4^+^ and CD8^+^ T cells in *n* = 6 autologous (auto.) and *n* = 12 allogeneic (allo.) combinations of Mregs and T cells (MW)

To assess the allospecificity of Mreg-cocultured T cell-mediated T cell-suppression, the following experiment was performed (Fig. 3a). Paired cultures of Mregs and mo-DCs were generated over 7 days from three unrelated donors: Mregs were then cocultured with allogeneic CD3^+^ T cells from a further two unrelated donors for 3 days leading to an enrichment of FoxP3^+^ T cells (25.9 ± 4.2%, *n* = 6 pairs). In parallel, mo-DCs were matured with TNFα. Subsequently, the capacity of CD3^+^ T cells re-isolated from Mreg cocultures to suppress allogeneic DC-driven proliferation of responder T cells (which were autologous to Mreg-cocultured T cells) was tested. Explicitly, re-isolated CD3^+^ T cells (not purified FoxP3^+^ T cells) were used as the suppressor population in these experiments; therefore, Mreg-induced FoxP3^+^ T cells represented only 8.1 ± 1.5% of all T cells initially present. Mreg-cocultured T cells suppressed both CD4^+^ and CD8^+^ responder T cells equally strongly when stimulator DCs were autologous or allogeneic to Mregs used in the initial coculture phase (Fig. 3b). That is to say, Mreg-cocultured T cells did not behave as allospecific suppressors in this experimental system. Nevertheless, it should be noted that FoxP3^+^ CD4^+^ T cells from Mreg cocultures are already activated and secrete IL-10 without further stimulation. Accordingly, these results show that freshly re-isolated Mreg-cocultured T cells mediate short-term, non-specific ‘bystander’ suppression, but they do not prove that initial activation of naive CD4^+^ T cells by Mregs is not allospecific or that Mreg-induced FoxP3^+^ CD4^+^ T cells would not suppress in an allo-restricted fashion in vivo.

### Phenotype of Mreg-cocultured CD4^+^ CD25^+^ FoxP3^+^ T cells

**Fig. 4:**
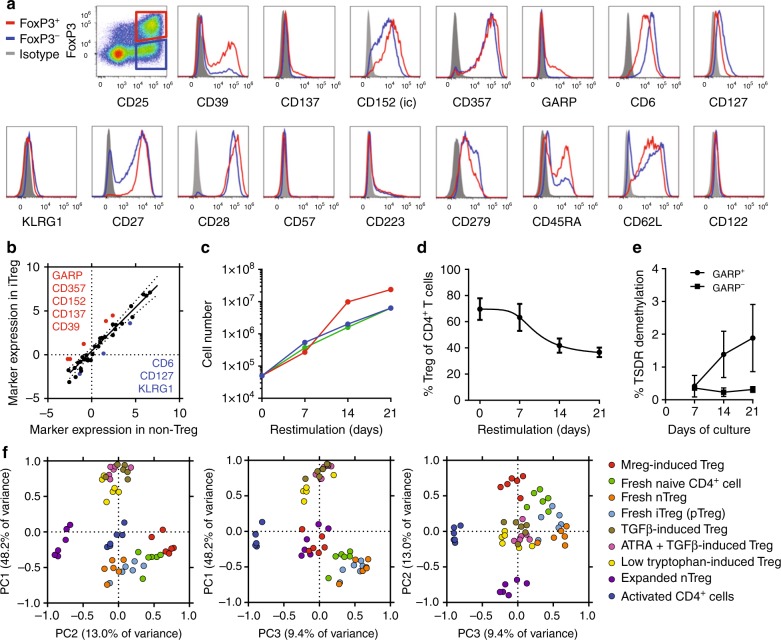
Mreg coculture enriches CD4^+^ T cells for GARP^+^ FoxP3^+^ iTregs. Marker screening experiments were performed in order to establish whether FoxP3^+^ and FoxP3^−^ T cells arising through Mreg coculture represented truly distinct populations. **a** Human Mregs were cultured at a 1:1 ratio with allogeneic human CD3^+^ T cells for 5 days before expression of Treg-associated markers was investigated by flow cytometry (representative of *n* = 6). **b** Correlation between marker expression in FoxP3^+^ miTregs and CD25^+^ FoxP3^−^ non-Tregs. Background (isotype)-subtracted MFI values were log_2_-transformed and median values for *n* = 6 donors were plotted. GARP, CD357 (GITR), intracellular CD152 (CTLA4), CD137 (4-1BB) and CD39 were more highly expressed by miTregs. CD6, CD127 and KLRG1 were more highly expressed by non-Tregs. **c** GARP-selected miTregs stimulated with αCD3/αCD28 beads expanded exponentially for 21 days after isolation (*n* = 3). **d** Over this 21-day re-stimulation with αCD3/αCD28 beads, frequencies of FoxP3^+^ miTregs decayed to 36.6 ± 5.2% (*n* = 3; mean ± sd). **e** No relevant increase in TSDR demethylation was detected during re-stimulation (*n* = 3; mean ± sd). **f** To better understand the relationship between miTreg and other in vitro-derived iTregs, a phenotypic comparison was made between nine CD4^+^ T-cell subtypes from *n* = 6 donors considering 29 markers measured by flow cytometry (Supplementary Figure 4D). Principal component (PC) analysis readily distinguished miTregs from other in vitro-derived iTregs, activated T cells and activated nTregs. This approach revealed miTregs were more similar to naive T cells and peripherally-induced Tregs (pTregs) than any other CD4^+^ T-cell subset

It is well-established that activation of conventional human CD4^+^ T cells induces transient expression of FoxP3 and other Treg markers; moreover, such activated effector cells can suppress proliferation of responder T cells in vitro through competitive or cytotoxic mechanisms that may not reflect their true regulatory activity. Therefore, we first sought to discriminate Mreg-induced FoxP3^+^ T cells from activated effector T cell subsets. An extensive survey was made of markers expressed by FoxP3^+^ and FoxP3^−^ CD25^+^ T cells from Mreg cocultures (Fig. 4a and Supplementary Figure 4A). FoxP3^+^ CD25^+^ T cells expressed higher levels of CD39, CD137 (4-1BB), intracellular CD152 (CTLA4), CD357 (GITR) and GARP than their FoxP3^−^ counterparts (Fig. 4a, b). Conversely, FoxP3^−^ CD25^+^ T cells expressed higher levels of CD6, CD127 (IL7RA) and KLRG1 than FoxP3^+^ T cells. Following Mreg exposure, FoxP3^+^ T cells expressed markers of T cell activation, which led us to ask whether they represented a subset of exhausted T cells or a type of differentiated memory T cell. Exhaustion and senescence of CD4^+^ T cells is associated with a CD27^low^ CD28^low^ CD57^+^ KLRG1^+^ CD223^+^ CD279^+^ phenotype; however, Mreg-enriched FoxP3^+^ T cells were CD27^high^ CD28^high^ CD57^−^ KLRG1^−/low^ CD223^−/low^ and CD279^+^ (Fig. 4a) so do not appear to be exhausted or senescent T cells. Human CD4^+^ T_EMRA_ cells are typically described as CD27^low^ CD28^low^ CD45RA^+^ CD62L^−^ CD122^+^ T cells; therefore, CD27^high^ CD28^high^ CD45RA^+/−^ CD62L^+^ CD122^−^ Mreg-enriched FoxP3^+^ T cells are not CD4^+^ T_EMRA_ (Fig. 4a and Supplementary Figure 4B). Accordingly, the properties of human Mreg-induced FoxP3^+^ T cells are most consistent with them being described as iTregs, as opposed to activated effector T cells, memory T cells or exhausted T cells. For brevity, ‘Mreg-induced iTreg’ is henceforth shortened to ‘miTreg’.

Normally, FoxP3 expression by in vitro-generated iTregs without demethylated TSDR can be easily extinguished by strong polyclonal activation^14^. To assess their phenotypic stability and proliferative potential, miTregs were isolated from allogeneic cocultures and then re-cultured. Because GARP was not detectably expressed in FoxP3^−^ T cells, but was expressed at a low level by most miTregs, it could be used as a surrogate marker for purification of viable cells (Supplementary Figure 4C). Exponential growth was observed when flow-sorted GARP^+^ miTregs were re-stimulated in culture using anti-CD3/CD28 beads plus exogenous IL-2 over a 3-week period (Fig. 4c). During this expansion, FoxP3 expression decayed from 69.6 ± 14.2 to 36.6 ± 5.2% (Fig. 4d). Although the proportion of FoxP3^+^ T cells declined as a consequence of re-stimulation, miTregs certainly did not disappear and their absolute number increased by >250-fold in all cases. No relevant increase in TSDR demethylation was detected during the re-stimulation phase (Fig. 4e) indicating that miTregs did not re-differentiate into nTreg-like cells and that miTregs were not supplanted by contaminating nTregs. Taken together, these results indicate miTregs represent a semi-stable state of T cell activation and are not mere transitory forms.

Various methods for in vitro generation of human FoxP3^+^ iTregs have been described^15^, including activation of naive CD4^+^ T cells in the presence of IL-2 and TGF-β, or activation under tryptophan-free conditions; however, such iTregs are phenotypically and functionally different from naturally occurring FoxP3^+^ Helios^−^ Tregs, which are often called peripheral (p)Tregs to emphasise this distinction^16^. To better understand how miTregs are related to other human iTregs^17,18^, pTregs, nTregs and non-Tregs, we assessed expression of 29 Treg-associated markers in nine comparator T cell populations by flow cytometry (Fig. 4f and Supplementary Figure 5). Background-subtracted mean fluorescence intensity was calculated for each marker for every cell type from six independent donors: these data were then reduced by principal component (PC) analysis. PC1 distinguished miTregs from other in vitro-derived iTregs, whereas PC2 and PC3 discriminated miTregs from activated T cells, fresh nTreg and expanded nTreg. In this analysis, miTregs were most similar to naive T cells and pTregs. Because miTregs exhibited many differences to other in vitro naive CD4^+^ iTregs, we infer that distinct mechanisms must contribute to their differentiation.

### Multiple non-redundant mechanisms of miTreg generation

**Fig. 5:**
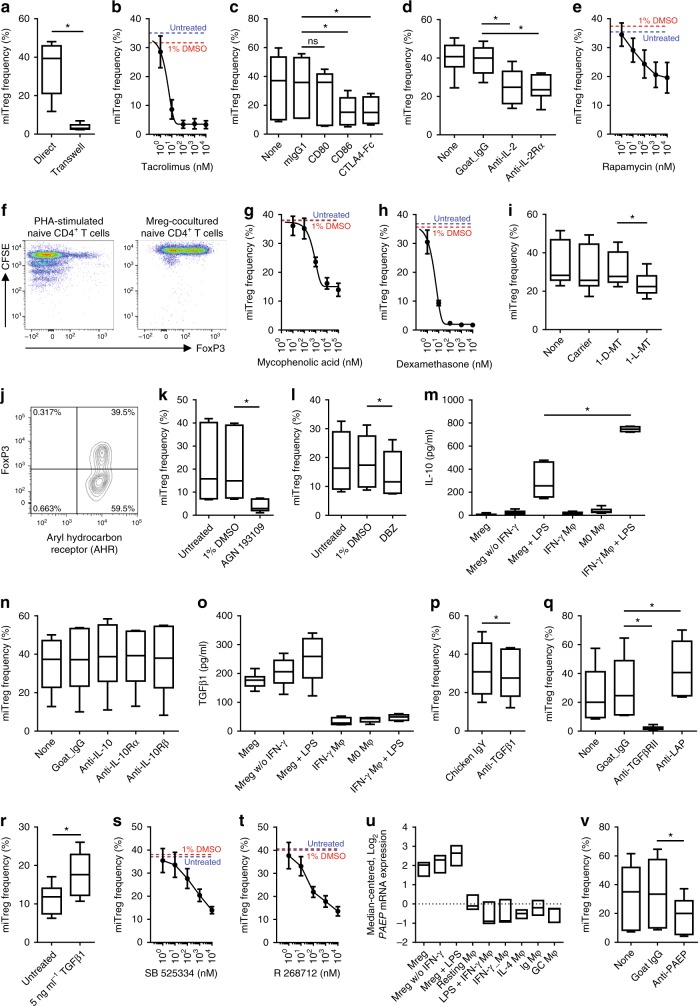
miTreg generation involves multiple non-redundant interactions. **a** miTreg did not arise when T cells and Mregs were separated using a transwell insert (*n* = 6; MW). **b** miTreg generation was inhibited in a dose-dependent manner by tacrolimus (*n* = 3; mean ± sd). **c** miTreg generation was inhibited by anti-CD86 or CTLA4-Ig, but not by anti-CD80 antibodies at 20 μg ml^−1^ (*n* = 6; KW). **d** miTreg generation was inhibited by 20 μg ml^−1^ anti-IL-2 or anti-IL-2Rα (*n* = 6; KW). **e** miTreg development was reduced by inhibiting mTOR signalling with rapamycin (*n* = 3; mean ± sd). **f** CFSE-labelled T cells did not proliferate during a 5-day coculture with allogeneic Mregs (*n* = 3). **g** miTreg generation was partly inhibited by mycophenolic acid (*n* = 3; mean ± sd). **h** miTreg generation was inhibited in a dose-dependent manner by dexamethasone (*n* = 3; mean ± sd). **i** miTreg generation was reduced when IDO was inhibited with 1 mM 1-l-methyltryptophan (1-l-MT) but not with 1 mM 1-d-MT (*n* = 6; MW). **j** Aryl hydrocarbon receptor (AHR) was expressed equally by FoxP3^+^ miTregs and CD25^+^ non-Tregs. **k** miTreg generation was inhibited with 10 μM AGN 193109, a pan-retinoic acid receptor antagonist (*n* = 6; MW). **l** miTreg generation was reduced when Notch signalling was blocked by inhibiting γ-secretase with 1 μM dibenzazepine (*n* = 6; MW). **m** Twenty-four-hour secretion of IL-10 by Mregs and comparator macrophages (*n* = 6). **n** Neutralising antibodies against IL-10, IL-10Rα and IL-10Rβ (each at 20 μg ml^−1^) did not affect miTreg generation (*n* = 6; KW). **o** Twenty-four-hour secretion of TGF-β_1_ by Mregs and comparator macrophages (*n* = 6). **p** A concentration of 20 μg ml^−1^ neutralising antibody against TGF-β_1_ consistently reduced miTreg generation (*n* = 9; paired *t*-test; *p* = 0.002). **q** miTreg generation was inhibited with 20 μg ml^−1^ anti-TGF-βRII and significantly enhanced by 20 μg ml^−1^ anti-LAP (*n* = 6; KW). **r** Addition of 5 ng ml^−1^ TGF-β_1_ to Mreg cocultures significantly increased miTreg generation (*n* = 6; MW). **s** miTreg generation was reduced in a dose-dependent manner by inhibiting TGF-βRI signalling with SB 25334 (*n* = 3; mean ± sd). **t** miTreg generation was reduced in a dose-dependent manner by inhibiting TGF-βRI signalling with R 268712 (*n* = 3; mean ± sd). **u** Relative expression of *PAEP* mRNA in a panel of nine human comparator macrophages (*n* = 3; mean ± sd). **v** miTreg generation was reduced by 20 μg ml^−1^ antibody against progestagen-associated endometrial protein (PAEP; *n* = 6; MW)

Enrichment of miTregs appears to be a specialised function of Mregs because IFN-γ Mφ do not have the same effect (Fig. 2h). Transwell separation of allogeneic Mregs and T cells in coculture demonstrated the need for direct physical interaction for Mreg-driven iTreg development (Fig. 5a). Inhibition of T cell receptor (TCR) signalling with tacrolimus significantly reduced miTreg induction (Fig. 5b). Blocking B7-mediated signalling with anti-CD86 or CTLA4-Ig showed that miTreg generation depended upon CD28 costimulation (Fig. 5c). Neutralisation of IL-2 or IL-2Rα reduced miTreg generation (Fig. 5d) and rapamycin also inhibited miTreg development at relatively high concentrations (Fig. 5e). Despite this role for IL-2 signalling, miTreg enrichment did not depend upon proliferation (Fig. 5f). Mycophenolic acid partly blocked miTreg development at concentrations above 100 nM (Fig. 5g), whereas 10 nM dexamethasone strongly suppressed miTregs (Fig. 5h). Because Mreg-mediated suppression of T cell proliferation is IDO-dependent, the effect of blocking IDO activity with 1-methyltryptophan (1-MT) was examined: 1 mM 1-l-MT significantly reduced miTreg development, whereas 1-d-MT had no effect (Fig. 5i). One mechanism by which IDO promotes Treg differentiation is degradation of tryptophan to kynurenines, which act as ligands for the aryl hydrocarbon receptor (AHR)^19^. No differential expression of AHR was detected between miTregs and non-Tregs (Fig. 5j). A pan-retinoic acid receptor antagonist (AGN 193109) effectively suppressed miTreg generation (Fig. 5k). Blocking Notch signalling with dibenzazepine, which acts as a γ-secretase inhibitor, impaired miTreg generation (Fig. 5l). Constitutive secretion of IL-10 by human Mregs was minimal and LPS-stimulated Mregs released significantly less IL-10 than classically activated macrophages (Fig. 5m). Neutralisation of IL-10, IL-10Rα or IL-10Rβ had no effect on miTreg development (Fig. 5n). Mregs constitutively produced more TGF-β_1_ than resting macrophages, IFN-γ Mφ or classically activated macrophages (Fig. 5o) and production was further enhanced by LPS stimulation. Neutralising TGF-β_1_ led to a consistent reduction in miTregs (Fig. 5p) and blocking antibody against TGF-βRII completely abrogated miTreg generation (Fig. 5q). Conversely, miTreg generation was enhanced by neutralising antibody against latency-associated peptide (Fig. 5q) or addition of exogenous TGF-β_1_ (Fig. 5r). Dose-dependent suppression of miTreg development was observed with two inhibitors of TGF-βRI signalling (Fig. 5s, t). From these experiments, it is evident that Mreg-mediated generation of iTregs involves multiple receptor-mediated and soluble factor-mediated interactions that are not unique to Mregs.

T cell activation, IDO activity, retinoic acid receptor ligation and TGF-β signalling were already known to promote in vitro differentiation of human iTregs; however, these factors alone do not account for the unique phenotype of miTregs (Fig. 4f). Therefore, we asked whether any Mreg-specific mechanisms contribute to miTreg differentiation. Transcriptional profiling studies comparing human Mregs to macrophages in other activation states identified genes highly and selectively upregulated in Mregs, including PAEP (alias PP14 or glycodelin) (Fig. 5u). In Mreg coculture experiments, antibody blockade of PAEP led to a significant reduction in miTreg generation (Fig. 5v). This example suggests that other undiscovered mechanisms might contribute to miTreg differentiation.

### Transcriptional profiling of Mreg-cocultured T cells

**Fig. 6:**
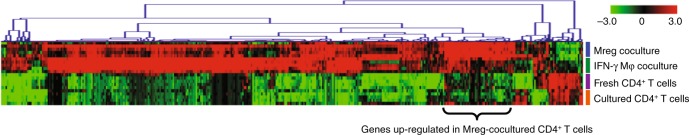
Identification of novel markers associated with miTregs. To better characterise the phenotype of miTregs, whole-genome gene expression-profiling studies by microarray were performed. Mregs and IFN-γ Mφ were generated from CD14^+^ monocytes of five healthy donors. Seven days later, CD3^+^ T cells were isolated from five unrelated donors and split into two lots: from the first lot, a pure population of fresh CD4^+^ T cells was obtained by flow-sorting and total RNA was extracted; the second lot of T cells were added into direct coculture with allogeneic Mregs or IFN-γ Mφ at a 1:1 ratio for 5 days, or were cultured alone without stimulation for 5 days. After culture, CD4^+^ T cells were flow-sorted and total RNA was extracted. A microarray data set was then generated that comprised four cell populations from five independent donors produced by single-colour hybridisations. The heatmap shows a hierarchical clustering of reporters returned by one-way ANOVA that were significantly (*p* < 0.01 after Benjamini-Hochberg correction for multiple testing) and highly differentially (>20-fold) regulated between any two of the T-cell populations (red: upregulation; green: downregulation)

To find novel pathways involved in miTreg development, whole-genome gene expression-profiling studies were conducted (Fig. 6). Mreg-cocultured and IFN-γ Mφ-cocultured CD4^+^ T cells were more similar to one another than to either fresh CD4^+^ T cells or CD4^+^ T cells cultured alone; however, substantial differences between Mreg- and IFN-γ Mφ-cocultured CD4^+^ T cells were returned by discriminatory gene analysis (Supplementary Data 1). Treg-associated genes were among those upregulated in Mreg-cocultured T cells compared to IFN-γ Mφ-cocultured T cells—notably, *FOXP3*, *IL2RA*, *EBI3* and *LRRC32* (*GARP*). *FOXP3* expression was upregulated by Mreg coculture, whereas key transcriptional regulators of Th1, Th2 and Th17 cell differentiation (ie. *TBET*, *GATA3* and *RORC*) were not differentially affected.

**Fig. 7:**
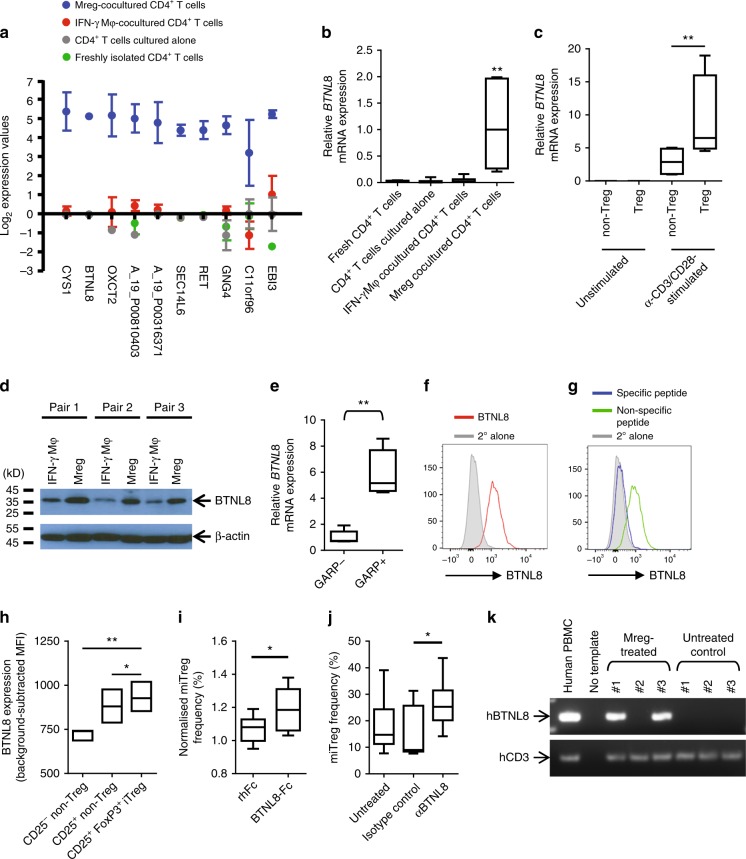
BTNL8 is expressed by miTregs. **a** Discriminatory gene analysis identified genes upregulated in Mreg-cocultured CD4^+^ T cells compared to controls. The 10 most highly upregulated reporters in allogeneic Mreg-cocultured CD4^+^ T cells compared to IFN-γ Mφ-cocultured CD4^+^ T cells are shown (*n* = 5; median ± MADM). **b** qPCR for *BTNL8* mRNA in CD4^+^ T cells cocultured with allogeneic Mregs or IFN-γ Mφ, CD4^+^ T cells cultured alone and freshly isolated CD4^+^ T cells (*n* = 9; mean ± SEM; KW). **c** Human peripheral blood T cells were flow-sorted for CD3^+^CD4^+^CD25^+^CD127^int^ Tregs and CD3^+^CD4^+^CD25^−^CD127^+^ non-Tregs. *BTNL8* mRNA expression was not detected in unactivated Treg or conventional T cells, but was induced by stimulation with αCD3/αCD28 beads for 5 days (*n* = 4; MW). **d** Western blot for BTNL8 using goat α-BTNL8 (Santa Cruz, sc-245053) to compare T cells cocultured with allogeneic Mregs or IFN-γ Mφ. A band corresponding to BTNL8 was detected at 37 kD. **e** *BTNL8* mRNA was more highly expressed by GARP^+^ miTregs than GARP^−^ non-Tregs (*n* = 6). **f** Detection of BTNL8 expression in Mreg-cocultured T cells by flow cytometry after intracellular staining with rabbit α-BTNL8 pAb (LSBio #C453018) against aa 161–210. Histogram is gated on live CD3^+^ CD4^+^ CD8^−^ CD25^+^ FoxP3^+^ single events. **g** Pre-incubation of rabbit α-BTNL8 pAb with specific peptide (LSBio #E18020) blocked detection of BTNL8, whereas a non-specific peptide corresponding to the N terminus of BTNL8 (#E18019) had no effect. **h** By flow cytometry, BTNL8 appeared to be expressed by all Mreg-cocultured CD4^+^ T cells, but miTregs expressed higher levels of BTNL8 than CD25^+^ FoxP3^−^ or CD25^−^ FoxP3^−^ non-Tregs (*n* = 3). **i** Compared to recombinant human Fc (rhFc), addition of 10 μg ml^−1^ BTNL8-Fc fusion protein enhanced miTreg generation (*n* = 8; MW). **j** A concentration of 20 μg ml^−1^ α-BTNL8 rabbit mAb (R&D #2187B) enhanced miTreg generation compared to isotype control (*n* = 9; MW). **k** NOD-scid-gamma (NSG) mice were reconstituted with 5 × 10^6^ naive human CD4^+^ T cells and then treated with either 5 × 10^6^ allogeneic Mregs (*n* = 3) or vehicle only (*n* = 3). Five days after cell transfer, splenic human T cells were recovered by bead-sorting, and end-point PCR for *BTNL8* mRNA was performed

One objective of this work was to identify markers and pharmacodynamic effects that could be used to infer direct interaction of donor-derived Mregs with recipient T cells after administration of Mregs to patients. To this end, we next examined genes that were highly and significantly upregulated by Mreg coculture (Fig. 7a). The 10 most discriminatory reporters included 5 genes encoding proteins with known function (*CYS1*, *OXCT2*, *RET*, *GNG4* and *EBI3*), one cell-surface receptor (butyrophilin-like protein 8; BTNL8), two predicted proteins of unknown function (*SEC14L6* and *C11orf96*) and one lincRNA (represented by reporters A_19_P00316371 and A_19_P00810403). Of these upregulated genes, *BTNL8* stood out as a possible marker of miTregs with likely functional relevance.

Upregulation of *BTNL8* mRNA after Mreg coculture was corroborated by quantitative PCR (qPCR) using primers that amplified both the BTN-like and B7-like variants of *BTNL8* (Fig. 7b). Using isoform-specific primer sets and sequencing of amplicons, it was determined that Mreg-cocultured T cells preferentially expressed the B7-like, ~37-kD variant of BTNL8. Expression of *BTNL8* mRNA was not detected in freshly isolated, human peripheral blood Tregs or conventional CD4^+^ T cells (Fig. 7c). However, *BTNL8* was highly expressed by peripheral blood Tregs after polyclonal stimulation and to a lesser extent in activated non-Tregs. Western blotting using goat α-BTNL8 pAb (Supplementary Figure 6) revealed stronger expression of the 37-kD isoform of BTNL8 in Mreg-cocultured T cells compared to IFN-γ Mφ-cocultured T cells (Fig. 7d). Expression of *BTNL8* mRNA was greater in GARP^+^ miTregs compared to GARP^−^ non-Tregs (Fig. 7e). BTNL8 protein was detected in miTregs by flow cytometry after intracellular staining with rabbit α-BTNL8 pAb (Fig. 7f, g). By this method, stronger expression of BTNL8 was found in miTregs than in CD25^+^ FoxP3^−^ or CD25^−^ non-Tregs (Fig. 7h). Addition of 10 μg ml^−1^ BTNL8-Fc fusion protein to allogeneic cocultures of Mregs and naive CD4^+^ T cells led to a small, but consistent increase in miTreg generation (Fig. 7i). Likewise, addition of 20 μg ml^−1^ anti-BTNL8 monoclonal antibody led to an increase in miTreg generation (Fig. 7j). Taken together, these results indicate that the 37-kD isoform of BTNL8 is more highly expressed in activated Treg than resting Treg or non-Treg, which raises the possibility that BTNL8 engagement promotes conversion of naive T cells to miTregs.

Reconstitution experiments were performed in NOD-scid-gamma (NSG) mice to ascertain whether in vivo administration of Mregs drives BTNL8 expression in allogeneic human T cells (Fig. 7j). All animals were given an i.v. injection of 5 × 10^6^ naive human T cells and were then randomised to receive a second i.v. injection of 5 × 10^6^ allogeneic Mregs or vehicle only. Human T cells were recovered from spleen on day 5 after treatment by positive bead selection of CD3^+^ cells and end-point PCR was performed to assess *BTNL8* expression. *BTNL8* was detected in two of three Mreg-treated recipients, but not in untreated recipients. We conclude that BTLN8 may be a valuable marker of human peripherally induced Tregs and could potentially be used for monitoring miTregs in patients.

### Mreg-induced Tregs express TIGIT

**Fig. 8:**
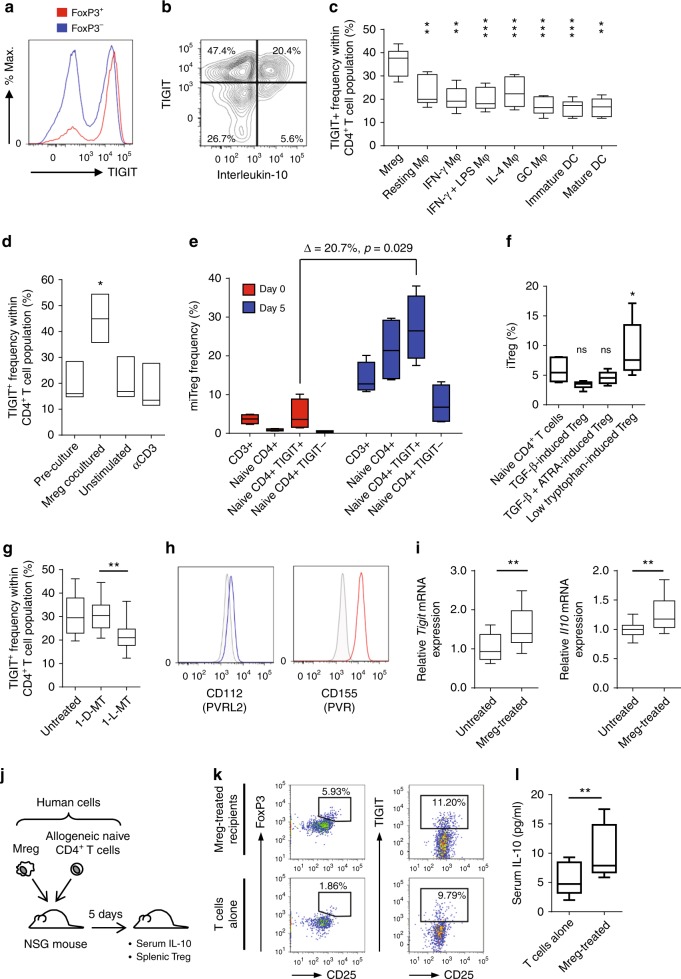
Mreg generation of IL-10-producing, TIGIT^+^ miTregs is IDO-dependent. Microarray data indicated higher expression of TIGIT (WUCAM, VSTM3) in Mreg-cocultured CD4^+^ T cells compared to control conditions. **a** Flow cytometry confirmed TIGIT expression by most miTregs and a high proportion of Mreg-cocultured non-Tregs (representative of *n* = 6). **b** After Mreg coculture, the majority of IL-10-producing CD4^+^ T cells were TIGIT^+^ (representative of *n* = 6). **c** Mreg coculture was significantly more effective in inducing TIGIT expression in CD4^+^ T cells than coculture with differently activated monocyte-derived macrophages or mo-DCs (*n* = 6; ANOVA). **d** TIGIT expression by CD4^+^ T cells was induced by Mreg coculture, but not by polyclonal T-cell activation with αCD3/αCD28 beads (*n* = 3; paired *t*-test). **e** Mregs preferentially convert naive CD4^+^ non-Tregs into miTregs (*n* = 6; MW). **f** Comparing different subsets of in vitro-derived human iTregs revealed highest TIGIT expression in low-tryptophan iTregs (*n* = 6; ANOVA) and suggested a role for IDO in Mreg-induced TIGIT expression. **g** Mreg-induced TIGIT expression was inhibited with 1 mM 1-l-methyltryptophan (1-l-MT) but not 1 mM 1-d-MT (*n* = 6; MW). **h** Mregs expressed TIGIT ligands CD155 (PVR) and CD112 (PVRL2). **i** BALB/c mice received either 5 × 10^6^ C3H strain Mregs by intravenous injection (*n* = 10) or vehicle only (*n* = 9). Five days later, splenic CD4^+^ T cells were isolated and expression of *Tigit* and *Il10* mRNA was assessed by qPCR. **j** Induction of TIGIT^+^ Tregs by allogeneic human Mregs in vivo was investigated using an immunodeficient (NSG) mouse model. NSG mice received either i.v. injection of 5 × 10^6^ human naive CD4^+^ T cells alone (*n* = 6) or i.v. injection of 5 × 10^6^ human naive CD4^+^ T cells plus a separate i.v. injection of 5 × 10^6^ allogeneic human Mregs (*n* = 6). After 5 days, serum levels of human IL-10 and splenic human TIGIT^+^ Treg frequencies were assessed. **k** Human Mreg-treated, T-cell-reconstituted NSG mice exhibited higher splenic Treg frequencies and somewhat higher TIGIT^+^ CD4^+^ T-cell frequencies. **l** Serum human IL-10 levels were significantly higher in human Mreg-treated, T-cell-reconstituted NSG mice than controls

Having identified BTNL8 as a possible marker of miTregs, we asked whether other BTN and BTNL family members were differentially regulated in these cells. No significant differences in expression of other BTN or BTNL family members were observed between Mreg-cocultured CD4^+^ T cells and controls (Supplementary Figure 7A). The microarray data set was then screened more broadly for differential expression of other IgSF co-inhibitory receptors. *T cell immunoreceptor with Ig and ITIM domains* (TIGIT) mRNA was more highly expressed in Mreg-cocultured T cells compared to control conditions (Supplementary Figure 7B). A greater proportion of miTregs expressed cell-surface TIGIT than non-Tregs (Fig. 8a). IL-10 production by Mreg-cocultured T cells was essentially restricted to TIGIT^+^ T cells (Fig. 8b). Mreg-mediated induction of TIGIT in CD4^+^ T cells was significantly more effective than TIGIT induction by other monocyte-derived macrophages and DCs (Fig. 8c). Expression of TIGIT by naive T cells was induced by Mreg coculture but not by anti-CD3/CD28 stimulation (Fig. 8d) so we next asked whether miTregs are predominantly TIGIT^+^ because they derive from pre-exisiting TIGIT^+^ non-Tregs. Prior to coculture with Mregs, naive CD4^+^ T cells were separated into TIGIT^+^ and TIGIT^−^ fractions by magnetic bead selection: TIGIT^+^ T cells gave rise to significantly more miTregs than either unfractionated naive T cells or TIGIT^−^ T cells (Fig. 8e). A greater proportion of low-tryptophan-induced iTregs expressed TIGIT compared to naive CD4^+^ T cells, TGF-β-induced iTregs or TGF-β plus all-*trans* retinoic acid (ATRA)-induced iTregs (Fig. 8f); therefore, we hypothesised that TIGIT induction by Mregs might be partly explained by IDO activity. Addition of 1-l-MT to cocultures, but not 1-d-MT, significantly reduced TIGIT^+^ T cell frequency (Fig. 8g). Mregs expressed both CD112 (PVRL2) and CD155 (PVR), which are ligands of TIGIT (Fig. 8h). These findings show that Mregs induce TIGIT expression in allogeneic T cells through an IDO-dependent mechanism, as well as providing TIGIT ligands, which might explain the high level of IL-10 production by miTregs.

To investigate whether allogeneic mouse Mregs induced TIGIT expression in CD4^+^ T cells in vivo, mouse Mregs were generated from C3H mice and administered by i.v. injection to BALB/c recipients at a dose of 5 × 10^6^ cells per animal. Five days after treatment, splenic CD4^+^ T cells were isolated by magnetic bead separation and reverse transcription (RT)-qPCR for *Tigit* and *Il10* mRNA was performed. Mice treated with allogeneic Mregs expressed significantly higher levels of both transcripts (Fig. 8i). A reconstitution experiment was then performed in NSG mice to test whether human Mregs could induce TIGIT and IL-10 expression by allogeneic human T cells in vivo (Fig. 8j). Human CD25^+^ FoxP3^+^ Treg frequencies were higher in Mreg-treated mice than vehicle-only controls, although proportions of TIGIT^+^ CD4^+^ T cells were similar (Fig. 8k). Serum levels of human IL-10 were significantly higher in Mreg-treated recipients compared to controls (Fig. 8l). These data indicate that allogeneic Mregs were capable of inducing TIGIT^+^ Tregs in this human-into-mouse model, which presumably involved direct allorecognition of Mregs by T cells.

### Acute increase of TIGIT^+^ Tregs after Mreg treatment

**Fig. 9:**
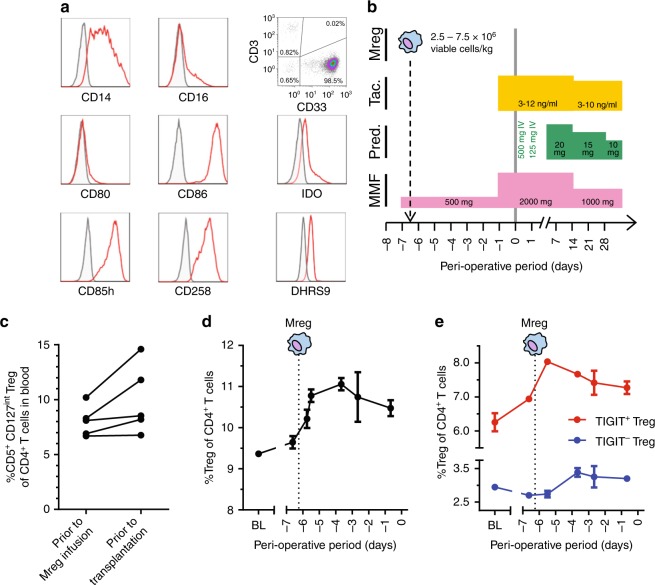
Changes in peripheral blood Treg numbers after Mreg_UKR administration. **a** An example of Mreg_UKR manufactured under clean-room conditions. For the pharmaceutical release of Mreg_UKR products, the identity of Mreg cells is specified according to CD14, CD16, CD80, CD86, CD85h and CD258 expression. It is specified that impurity of Mreg_UKR products with CD3^+^ T cells must be ≤1%. It is further specified that Mreg cells must upregulate IDO expression as a marker of potency. DHRS9 expression is not specified as a product-release criterion; however, Mreg cells contained in Mreg_UKR products uniformly express DHRS9. **b** One week prior to living-donor kidney transplantation, patients in the *ONEmreg12* trial are treated with a single dose of 2.5–7.5 × 10^6^ viable Mregs per kg by central i.v. infusion under cover of mycophenolate mofetil (MMF) 250 mg BD. One day prior to kidney transplantation, patients receive MMF 1 g BD and tacrolimus (Tac) treatment commences at a target serum trough level of 3–12 ng ml^-1^. Patients receive an intraoperative steroid bolus, but no basiliximab (anti-IL-2Rα) induction therapy. **c** CD25^+^ CD127^int^ Treg frequencies in peripheral blood of Mreg-treated patients assessed prior to Mreg infusion and 1 day before transplantation. Results from five patients are shown, including two participants of the *TAIC-II Study* whose cases were previously reported elsewhere. **d** In the case of patient C3157, serial blood samples were collected for measurement of CD4^+^ CD25^+^ FoxP3^+^ Tregs prior to Mreg infusion and at close intervals over the 6 days before transplantation (values represent mean ± sd of replicate stainings). **e** The observed increase in peripheral blood Treg frequency in patient C3157 could be mainly attributed to an increase in TIGIT^+^ Tregs

As early as 2008, prospective living-donor kidney transplant recipients were given crude Mreg preparations in the expectation of reducing long-term dependence on immunosuppressive drug treatment^10^. These first-in-man cases demonstrated the clinical feasibility of Mreg treatment in kidney transplantation, yielded important safety information and provided hints of a beneficial immunological effect. Following significant technical refinements, a good manufacturing practice-compliant production process for manufacturing an Mreg-containing cell product, which is known as ‘Mreg_UKR’, was established (Fig. 9a)^11^. Mreg_UKR is under investigation as a means to safely minimise maintenance immunosuppression after kidney transplantation in the *ONEmreg12* trial. This phase-I/II study provides an opportunity to study the influence of allogeneic Mreg treatment upon recipient T cell responses. According to protocol, *ONEmreg12* trial participants are treated with donor-derived Mregs on day 7 prior to transplantation (Fig. 9b). In the peri-operative phase, patients received standard-of-care, triple immunosuppressive therapy, except basiliximab (anti-IL-2Rα) induction therapy is omitted.

Altogether Treg frequencies were monitored in five allogeneic donor–recipient pairs before Mreg infusion and 1 day prior to living-donor kidney transplantation, including two patients recruited to *ONEmreg12* and three participants of earlier clinical trials^10^. All five patient pairs were mismatched at one or more HLA-A, -B or -DR locus. In four out of five patients, an increase in peripheral blood CD4^+^ CD25^+^ CD127^int^ Treg frequency was observed (Fig. 9c). A single patient enrolled in the *ONEmreg12* trial presented an opportunity to assess peripheral blood frequencies of TIGIT^+^ Tregs at close intervals during the week after Mreg infusion. This high-granularity sampling revealed an increase in Treg frequency during the first 18 h after Mreg infusion, which peaked by the third day (Fig. 9d). Notably, this increase was primarily due to an increase in TIGIT^+^ Treg frequency (Fig. 9e). This first description of TIGIT^+^ Treg induction after Mreg treatment must be confirmed in more patients; however, it is promisingly consistent with our preclinical experiments.

### Long-term enrichment of TIGIT^+^ Tregs after Mreg therapy

**Fig. 10:**
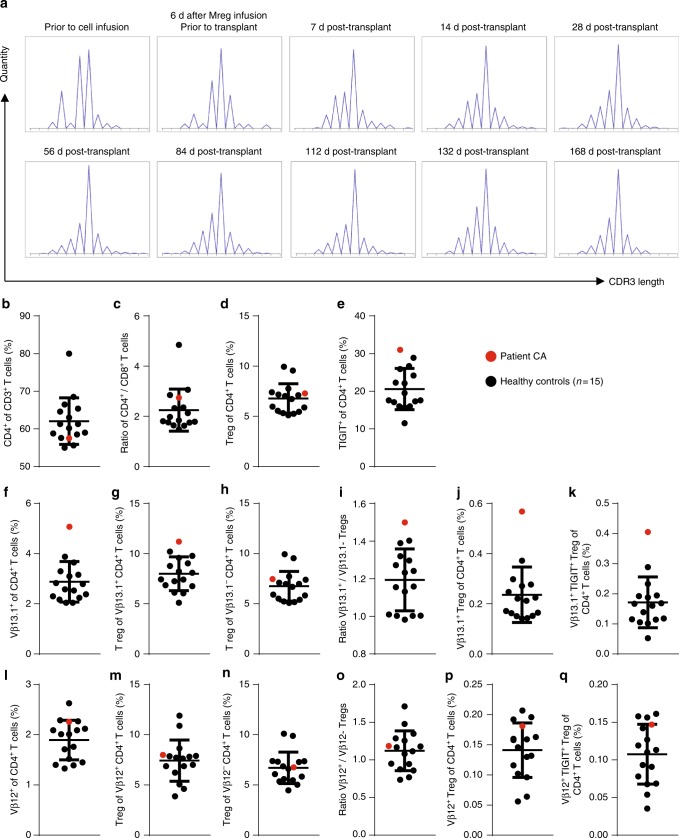
Elevated frequency of TIGIT^+^ TCRVβ13.1^+^ FoxP3^+^ Tregs in the peripheral blood of an Mreg-treated kidney transplant recipient at ~7 years after surgery. The 1-year outcome of an Mreg-treated kidney transplant recipient, who is known as patient CA, has been previously reported. At ~7 years post transplant, patient CA had stable graft function and was receiving tacrolimus monotherapy as his only maintenance immunosuppression. **a** At intervals during the first months of follow-up, peripheral blood samples were taken from patient CA for PCR-based TCR spectratyping. These analyses revealed an oligoclonal expansion of TCRVβ13.1^+^ T cells that occurred after Mreg administration and persisted throughout the first 6 months. **b–q** At ~7 years post transplant, peripheral blood samples from patient CA were analysed by flow cytometry for the presence of TCRVβ13.1^+^ T cells. Compared to 15 healthy donors without transplants, patient CA was not exceptional with respect to **b** CD4^+^ T-cell frequency, **c** CD4^+^/CD8^+^ T-cell ratio or **d** overall frequency of FoxP3^+^ Treg. **e** Patient CA exhibited only a marginally higher frequency of peripheral blood TIGIT^+^ CD4^+^ T cells compared to the reference population. **f** Patient CA exhibited a higher frequency of TCRVβ13.1^+^ T cells in peripheral blood than controls. **g** Patient CA had a higher proportion of Tregs within TCRVβ13.1^+^ CD4^+^ T cells compared to his overall CD4^+^ T-cell pool. **h** By contrast, Tregs were not enriched amongst TCRVβ13.1^−^ CD4^+^ T cells. **i** Patient CA showed a higher ratio of TCRVβ13.1^+^ Treg to TCRVβ13.1^−^ Treg than healthy controls. **j** As a proportion of all CD4^+^ T cells, TCRVβ13.1^+^ Treg frequency was higher in patient CA than healthy controls. **k** Notably, patient CA also exhibited a higher proportion of TCRVβ13.1^+^ TIGIT^+^ Treg within the CD4^+^ T-cell compartment compared to the reference group. **l** Consistent with TCR spectratyping results from the first 6 months, patient CA exhibited no enrichment of TCRVβ12^+^ T cells. **m**–**q** Differences between patient CA and healthy controls observed in TCRVβ13.1^+^ CD4^+^ T cells were not reflected in TCRVβ12^+^ T cells

Serial TCR spectratyping was performed in one patient (CA) after treatment with Mregs^10^. This led to identification of an oligoclonal or monoclonal expansion of TCRVβ13.1-expressing T cells that first emerged after Mreg treatment and persisted until at least 168 days post transplant (Fig. 10a). The kinetics of this T cell expansion suggested a donor alloantigen-specific response. CA was followed-up at ~7 years post transplantation, when graft function was stable and no rejection episodes had occurred under very low-dose tacrolimus monotherapy (3 mg Advagraf every second day) with trough levels of <2 ng ml^−1^. Expansion of TCRVβ13.1^+^ T cells was still detectable at the time of follow-up and, more surprisingly, we found the TCRVβ13.1^+^ CD4^+^ T cell compartment was enriched for TIGIT^+^ FoxP3^+^ Tregs. These data are interpreted as evidence of a donor-specific expansion and long-term persistence of TIGIT^+^ TCRVβ13.1^+^ Tregs.

## Discussion

Cell-based tolerance-promoting therapies are a radical departure from conventional immunosuppressive treatment of solid organ transplant recipients^1^. As with any drug treatment, understanding the pharmacological character of cell-based therapies is vital for predicting effectiveness and anticipating adverse reactions^20^. In this study, Mregs converted a high proportion of directly cocultured, allogeneic CD4^+^ T cells into IL-10-secreting miTregs. These miTregs were not simply activated T cells, exhausted T cells^21^ or T_EMRA_. miTregs did not derive from nTreg because they arose in Treg-depleted naive CD4^+^ T cell cultures and TSDR demethylation was minimal^22^. Administration of mouse Mregs to allogeneic recipients increased *Tigit* and *Il10* mRNA expression by splenic CD4^+^ T cells in vivo. In a human-into-mouse reconstitution model, human Mregs also converted allogeneic CD4^+^ T cells to IL-10-producing iTregs. These preclinical observations led to the discovery that Mreg treatment of prospective kidney transplant recipients elicited TIGIT^+^ Treg responses. Overall, our study supports the idea that miTreg could play a mechanistic role in the clinical effects of Mreg therapy.

miTreg development involves multiple, non-redundant interactions that are not necessarily specific to Mregs, but together confer Mregs with specialised regulatory function. We found a prominent role for IDO in miTreg differentiation and TIGIT expression, but this does not sufficiently explain the iTreg-inducing capacity of Mregs. In our system, miTreg differentiation required T cell activation and was influenced by TGF-β, RA, PAEP and BTNL8 signalling. Looking for further mechanisms, we annotated reporters upregulated in Mreg-cocultured CD4^+^ T cells according to Gene Ontology pathways (Supplementary Data 2). This revealed enrichment of genes associated with Notch signalling^23^. Using a γ-secretase inhibitor, the involvement of canonical Notch signalling was confirmed. Beyond these verified mechanisms, expression-profiling data suggest possible involvement of vitamin D, arginase and negative costimulatory pathways in miTreg development, stabilisation or survival. Hence, the regulatory activity of Mregs cannot be explained by a single, Mreg-specific mechanism: it reflects the net effect of many pathways, some of which may be common to other myeloid APCs. It has not escaped our attention that Mreg:T cell coculture could be used as a functional screening assay to discover novel mechanisms of iTreg development or identify drugs that influence iTreg differentiation^24^.

PAEP, which is also known as glycodelin, is a 28-kDa glycoprotein produced by secretory and decidualised endometrium in women and by seminal vesicle epithelium in men. PAEP plays critical roles in placental development and establishment of fetomaternal tolerance in early pregnancy^25^. Specifically, PAEP controls the number and effector functions of decidual T cells, favouring a Th2-type environment through selective apoptosis of Th1 cells^26^. Within the haematopoietic system, PAEP is expressed by erythroid precursors, but not by mature erythrocytes, thrombocytes, mononuclear phagocytes or lymphocytes^27^. PAEP secretion is presently the only known mechanism of miTreg induction exclusive to Mregs.

This study discovered the B7-like isoform of BTNL8 as potentially useful marker of activated miTregs. BTNL8 is an intriguing receptor from a basic immunological perspective^28^. The butyrophilin (BTN) and butyrophilin-like (BTNL) proteins are B7-related receptors that serve as negative regulators of innate and adaptive immune responses^29^. The human genome encodes four BTNL genes (*BTNL2*, *-3*, *-8* and *-9*) of which only *BTNL2* and *BTNL9* have orthologues in mice^30^. Ig-fusion proteins of mouse Btnl1, Btnl2 and Btnl9 exert T cell-suppressive properties in vitro through unknown counter-receptors^31–33^; however, functional characterisation of human BTNL family members is sparse. BTNL8 is a type-I membrane protein with an extracellular region comprising an IgV and IgC domain^28^. Various isoforms of BTNL8 have been reported, including a 57-kD protein incorporating an intracellular B30.2 domain, which is lacking from the 37-kD isoform. Expression of both variants in enterocytes^34^, neutrophils and eosinophils^35^ has been reported, but not T cells, B cells, natural killer (NK) cells or monocytes^30,36^. Here the 37-kD isoform of BTNL8 was highly upregulated in Mreg-cocultured CD4^+^ T cells, especially the GARP-selected miTreg-enriched fraction. Recently, a human BTNL8-Fc fusion protein was shown to bind an unknown receptor on resting T cells that co-stimulated anti-CD3-induced activation^35^. In our experiments, BTNL8-Fc and α-BTNL8 antibody increased miTreg generation. We hypothesise that miTregs stimulate bystander naive T cells through BTNL8, thereby amplifying iTreg generation.

TIGIT is a co-inhibitory receptor of the CD28 family^37^ that is expressed by NK cells^38^, activated T cells, memory T cells^39^, follicular T helper cells^40^ and Treg subsets^41–43^. Mregs strongly induced TIGIT^+^ T cells through a partly IDO-dependent mechanism. The *TIGIT* gene is a direct target of FoxP3, which accounts for TIGIT expression in nTregs, but does not explain how TIGIT expression could precede FoxP3 induction in Mreg-cocultured T cells. TIGIT competes against the costimulatory receptor CD226 (DNAM-1)^44^ for ligation by CD155 or CD112^45^. After Mreg coculture, the proportion of CD226^+^ TIGIT^+^ iTregs was higher than CD226^−^ TIGIT^+^ iTregs, but the functional significance of this is unknown. Engagement of TIGIT on conventional T cells delivers an ITIM-mediated signal that blocks T cell proliferation and effector functions^46^ by downregulating components of the TCR signalling cascade^47^. At the same time, TIGIT upregulates anti-apoptotic molecules and receptors for IL-2, IL-7 and IL-15, thereby promoting survival of functionally anergised T cells. In nTregs, TIGIT engagement upregulates IL-10 production and Fgl2 secretion, which preferentially suppresses Th1 and Th17 responses^48^. IL-10 expression by Mreg-cocultured T cells closely correlated with TIGIT expression. CD155 ligation by TIGIT delivers tolerising signals to APC^49^, which might contribute to the stability of Mreg regulatory function^50^. Increased TIGIT^+^ Treg frequency in Mreg-treated patients speaks to the likely importance of TIGIT for the clinical effects of Mreg therapy.

In theory, donor-derived Mregs might influence antigen-specific recipient T cell responses in two main ways, which are not mutually exclusive^51^: first, Mregs might give up alloantigen^52^ to recipient APC, which then modify recipient T cell responses^3^; second, infused Mregs might interact directly with recipient T cells to anergise or delete antigen-specific effector T cells^53^ or induce Tregs^54^. The tolerogenic properties of i.v. alloantigen are well-known^55,56^ and it seems probable that reprocessing of Mreg-derived antigens by recipient APC (via indirect or semi-direct pathways) contributes to the therapeutic benefit of Mreg treatment. The suggestion that Mregs might also interact directly with recipient T cells to suppress or regulate their reactivity^57^ is controversial because others have shown that recipient cDC are indispensible for the tolerising effect of donor-derived immature DC treatment in some systems^58^. Notably, Wang et al. used CD11c-DTR bone marrow chimeras to show that pre-transplant administration of Vit.D_3_-treated, ‘maturation-resistant’ DC (MRDC) did not prolong allograft survival after deletion of recipient DC^59^. In this model, donor-strain MRDCs were quickly eliminated by NK cells, leading to their uptake by recipient DCs as apoptotic remnants. Alloantigen-loaded recipient DCs then controlled alloreactive T cell responses through abortive activation.

It is clear that all myeloid APC are not equally susceptible to rejection through innate and adaptive mechanisms^60,61^. Tolerance of solid organ transplants is often associated with long-term persistence of donor-derived mononuclear phagocytes in lymphoid and non-lymphoid tissues of the recipient^57,62–67^. Whether these residual DCs and macrophages contribute to allograft tolerance or are a mere epiphenomenon is not resolved; however, their engraftment implies escape from rejection. Earlier cell-tracking experiments in immunocompetent mice showed survival of allogenic and isogeneic Mregs in non-immunosuppressed recipients was very similar^12^, so it seems rejection did not limit the lifespan of Mregs. Likewise, in humans, HLA-haploidentical Mregs administered by central i.v. infusion remained viable over at least 30 h^10^. Because a detectable fraction of ex vivo-generated allogeneic Mregs migrates to, and survives for days or weeks, at sites where interaction with recipient T cells could conceivably occur then there is no a priori reason why donor-derived Mregs could not initiate direct-pathway recipient iTreg responses.

Even a transient expansion of allospecific iTregs could bias recipient T cell responses in favour of allograft acceptance. We imagine that miTregs could contribute to long-term acceptance of allografts in two ways. First, direct iTregs could facilitate indirect nTreg responses, perhaps by modifying recipient DC^68,69^ to initiate a feed-forward loop of ‘infectious tolerance’^70^. Notably, immature mo-DCs exposed to Mreg-cocultured T cells were relatively refractory to maturation with TNFα. The capacity of these ‘tolerised’ DC to support a second wave of Tregs is currently being investigated. A second possibility is that miTregs survive long term and continue to exert a suppressive activity. This hypothesis is supported by the surprising discovery of persistently elevated of Vβ13.1^+^ TIGIT^+^ Treg numbers in patient CA. Recent reports indicate that peripherally induced FoxP3^+^ Tregs can be stabilised in phenotype and function by TSDR region demethylation. We are currently exploring whether TSDR demethylation in miTregs can occur in vitro or in vivo.

As a translational therapy, understanding the possible interactions of therapeutically applied Mregs with recipient T cells is crucial. The new mechanistic insights presented in this article provide a pharmacological rationale for applying Mreg_UKR therapy in allogeneic solid organ transplantation and other T cell-mediated diseases.

## Methods

### Generation of human monocyte-derived cells

Human Mregs and other monocyte-derived cell types were generated from CD14^+^ monocytes according to previously described methods^7^. Step-by-step protocols are available through Protocol Exchange^71^. Human Mregs and IFN-γ Mφ were generated from peripheral blood leucocytes obtained as a by-product of thrombocyte collection from healthy donors. CD14^+^ monocytes were then isolated from Ficoll-prepared peripheral blood mononuclear cells by positive selection with anti-CD14 microbeads (Miltenyi, Bergisch-Gladbach) and were plated in six-well Cell+ plates (Sarstedt, Nümbrecht) at 10^6^ cells per well in RPMI-1640 (Lonza, Cologne) supplemented with 10% heat-inactivated, male-only human AB serum (Lonza), 2 mM Glutamax (Invitrogen, Karlsruhe), 100 U ml^−1^ penicillin (Lonza), 100 μg ml^−1^ streptomycin (Lonza), and recombinant human M-CSF (rhM-CSF; R&D Systems, Wiesbaden-Nordenstadt) at 25 ng ml^−1^ carried on 0.1% human albumin (CSL-Behring, Hattersheim-am-Main). On day 6 of culture, cells were stimulated for a further 18–24 h with 25 ng ml^−1^ rhIFNγ (Chemicon, Billerica, MA). IFN-γ Mφ were generated by cultivating CD14^+^ monocytes under identical conditions to Mregs except that human serum was replaced with 10% heat-inactivated foetal calf serum (Biochrom, Berlin). Step-by-step protocols for generating other human monocyte-derived macrophages and mo-DCs are available through Protocol Exchange^71^. Phagocytosis of pHrodo Red-labelled, fixed *E. coli* by Mregs and classically activated macrophages was quantified at intervals over a 9-h period according to the manufacturer’s instructions (ThermoFisher Scientific, CA).

### Coculture of human Mregs or IFN-γ Mφ with T cells

On day 7 of culture, Mreg and IFN-γ Mφ medium was exchanged with serum-free X-VIVO 10 medium (Lonza) supplemented with 2 mM Glutamax and 25 ng ml^−1^ rhM-CSF. Magnetic bead-sorted CD3^+^ T cells (CD3 Microbeads, Miltenyi) or naive CD4^+^ T cells (Naive CD4^+^ T Cell Isolation Kit II, Miltenyi) were added to macrophages at specified suppression:responder ratios for 5 days. Cocultures were performed either in six-well Cell+ plates at 10^6^ Mreg in 3 ml per well or in sterile, lidded flow cytometry tubes (Falcon, Amsterdam) at 2 × 10^5^ Mreg in 1 ml per tube. Inserts with 0.4 μm pores were used for transwell experiments (Greiner-BioOne, Frickenhausen). Pharmacological inhibitors were added to cocultures as indicated (Supplementary Table 1).

### In vitro-derived human iTregs and expanded human nTregs

Following protocols adapted from the literature, three alternative preparations of in vitro-derived human iTregs were differentiated from bead-sorted human CD4^+^ naive T cells (Naive CD4^+^ T Cell Isolation Kit II, Miltenyi) grown in round-bottomed 96-well plates at 1.2 × 10^5^ cells per well. ‘Low-tryptophan iTregs’ were generated over 5 days in tryptophan-free medium (PAN-Biotech, Aidenbach, Germany) supplemented with 10% dialysed heat-inactivated human AB serum, 100 U ml^−1^ penicillin, 100 µg ml^−1^ streptomycin, 4 µM tryptophan (Sigma-Aldrich) and 300 IU IL-2 ml ^−1^ (Miltenyi)^18^. On day 0, 100 µl cultures were stimulated with CD3/CD28 MACSi-beads (Miltenyi) and 10 µM each of l-kyneurinine, quinolic acid/2,3 pyridinedicarboxilyc acid, anthranilic acid, 3-hydroxy-anthranilic acid and 3-hydroxy-dl-kyneurinine (Sigma-Aldrich). On day 2, 100 µl supplemented medium containing 20 µM each of the same mixture of kynurenines was added per well. ‘TGF-β-induced iTregs’ were generated in X-Vivo 10 medium (Lonza) supplemented with 2 mM GlutaMax, 300 IU IL-2 ml ^−1^ and 5 ng ml^−1^ TGF-β_1_ (R&D Systems)^17^. On day 0, 100 µl cultures were stimulated with CD3/CD28 MACSi-beads. On day 2, 100 µl supplemented medium was added to each well. ‘ATRA + TGF-β-induced iTregs’ were cultured as TGF-β-induced iTregs except that medium was also supplemented with 10 nM ATRA (Sigma-Aldrich)^17^. Expanded human nTregs were generated from bead-sorted CD4^+^ CD25^+^ T cells (CD4^+^CD25^+^ Regulatory T Cell Isolation Kit, Miltenyi) cultured in X-Vivo 10 medium supplemented with 2 mM GlutaMax and 300 IU IL-2 ml ^−1^. On day 0, 100 µl cultures were stimulated with CD3/CD28 MACSi-beads. On day 2, 100 µl supplemented medium was added to each well. ‘Activated CD4^+^ T cells’ were generated from bead-sorted naive CD4^+^ T cells cultured in X-Vivo 10 medium supplemented with 2 mM GlutaMax and 300 IU IL-2 ml ^−1^. On day 0, 100 µl cultures were stimulated with CD3/CD28 MACSi-beads. On day 2, 100 µl supplemented medium was added to each well.

### Flow cytometry

Detailed, step-by-step protocols for flow cytometry-based analyses are available through Protocol Exchange^72^. Briefly, surface staining was performed at 4 °C in DPBS/1% BSA/0.02% NaN_3_/10% FcR-block (Miltenyi) for 30 min. FoxP3 Fixation-and-Permeabilization buffers (eBioscience, Frankfurt) were used for intracellular staining. Antibodies are listed in Supplementary Table 2. Dead cells were excluded with Fixable Viability Dye eFluor506 or 7-AAD (eBioscience). Data were collected with a Canto-II cytometer (BD Biosciences) running Diva version 6.1.3 (BD) or Navios cytometer (Beckman Coulter, Krefeld, Germany). Clinical data were collected with a qualified Navios cytometer running Beckman Coulter clinical software version 1.3. Analyses were performed with Flowo (v7.6.5) or Kaluza 1.1 (Beckman Coulter).

### Western blotting and immunoprecipitation

Protein G-Plus sepharose (Sigma-Aldrich) was used to immunoprecipitate BTNL8. SDS-polyacrylamide gel electrophoresis and immunoblotting were performed according to conventional methods. Uncropped western blotting images were included in Supplementary Figure 8.

### TSDR methylation analysis

CD4^+^ T cells were positively isolated by magnetic-activated cell sorting. Genomic DNA was extracted with the QIAamp DNA blood mini-kit and bisulfite-treated (EpiTect, Qiagen) before real-time PCR quantification of Foxp3 TSDR.

### Quantitative reverse transcription-PCR

SuperScript-III (Invitrogen) was used for reverse transcription. Quantitative reverse transcription-PCR was performed with a LightCycler™ real-time PCR system using the FastStart DNA Master SYBR Green I kit (Roche-Diagnostics, Penzberg) and Quantitect primers (Qiagen) listed in Supplementary Table 3. Gene expression was normalised against expression of *RPL13A* and *GAPDH*. PCR specificity was confirmed by amplicon sequencing (MWG-Biotech, Ebersberg).

### Microarrays

Highly purified populations of CD4^+^ or CD8^+^ T cells were isolated using a FACS-Aria flow-sorter (BD Biosciences). Total RNA was extracted using an RNeasy-Plus Mini-Kit (Qiagen, Hilden). Assessment of RNA quality, labelling and hybridisation to 8 × 60K Agilent Whole-Human-Genome-Oligo-Microarrays was performed according to published methods^12^. Fluorescence signals from hybridised microarrays were detected using Agilent’s Microarray Scanner System (G2505C, Agilent-Technologies, Palo Alto). The Agilent Feature Extraction Software 10.7.3.1 was used to extract the microarray image files. Background-corrected intensity values were quantile-normalised and log_2_-transformed. Only reporters with at least three valid signal intensity values in at least one sample group were considered. For visualisation in heatmap format, the log_2_ intensity values were median-centred for each reporter. One-way analysis of variance (ANOVA) was performed using GeneSpring-GX v.11.5.1 (Agilent).

### Statistics

Significance tests and curve-fitting were performed with GraphPad or SPSS^®^ software. As indicated, Mann–Whitney, Kruksal–Wallis, *t*-tests (two-tailed, equal variances; paired where appropriate) or one-way ANOVA was used for tests of significance. Marker expression was estimated in flow cytometry experiments by calculating background (isotype)-subtracted geometric mean fluorescence intensities. Values ≤ 0 were set to 1. Data were log_10_-transformed and expression of each marker was standardised. PC analysis with oblique rotation (oblimin) was performed in SPSS^®^ version 25.

### Animal experiments

Animal experiments were performed with the approval of the Regierung der Oberpfalz in accordance with the German Animal Welfare Act, approval 54-2332.1-23/10 and approval 54-2532.1-10/12. Mouse Mregs were generated from 8- to 12-week-old C3H mice and administered to 8- to 12-week-old BALB/c recipients according to previously published methods^12^. NSG (NOD.Cg-*Prkdc*^*scid*^ *Il2rg*^*tm1Wjl*^/SzJ) mice were bred in-house and were used at 8–12 weeks of age. For administration to mice, human and mouse Mregs were resuspended in 1 ml DPBS containing 62 U heparin and then delivered by slow injection into the tail vein. Animals were randomly allocated to treatment or control groups by a blinded operator.

### Study approval

Mreg_UKR products were administered to consenting kidney transplant recipients who were participating in the *ONEmreg12* trial (clinicaltrials.gov: NCT02085629) authorised by the Paul Ehrlich Institute (EudraCT-Nr. 2013-000999-15) and the Ethics Committee of University Hospital Regensburg (14-111-0016). *ONEmreg12* is a non-commercial, investigator-initiated, monocentre, single-arm, phase-II trial operating within the framework of ONE Study cell therapy trials (www.onestudy.org). Collection of mononuclear cell apheresates by the Department of Transfusion Medicine at University Hospital Regensburg was authorised by the Regierung der Oberbayern under license DE_BY_04_MIA_2013_0177/53.2-ZAB-2677.1_204. Production of Mreg_UKR by a contract manufacturing organisation was authorised by the Regierung der Oberbayern under license DE_BY_04_MIA_2013_0187/53.2-2677.1_A_220-0. This article includes results from three patients treated according to a modification of the *TAIC-II Study* protocol (ClinicalTrials.gov: NCT00223067), including two patients whose cases were previously reported elsewhere.

### Data availability

The gene expression data presented in this article have been submitted to the National Center for Biotechnology Information/Gene Expression Omnibus as data set GSE49369. All other relevant data are available from the authors without restriction.

## Electronic supplementary material

Supplementary Information

Description of Additional Supplementary Files

Supplementary Data 1

Supplementary Data 2
