# Supplementary material for: TIGIT+ iTregs elicited by human regulatory macrophages control T cell immunity
Source: Nat Commun. 2018 Jul 20;9:2858. doi: 10.1038/s41467-018-05167-8 (PMC6054648; doi:10.1038/s41467-018-05167-8)
Supplement: Supplementary file 1 — Supplementary Information [file 41467_2018_5167_MOESM1_ESM.pdf]

Supplementary Information

Riquelme et al., 2018

TIGIT^{+} iTregs elicited by human regulatory macrophages control T cell immunity

SUPPLEMENTARY FIGURE 1

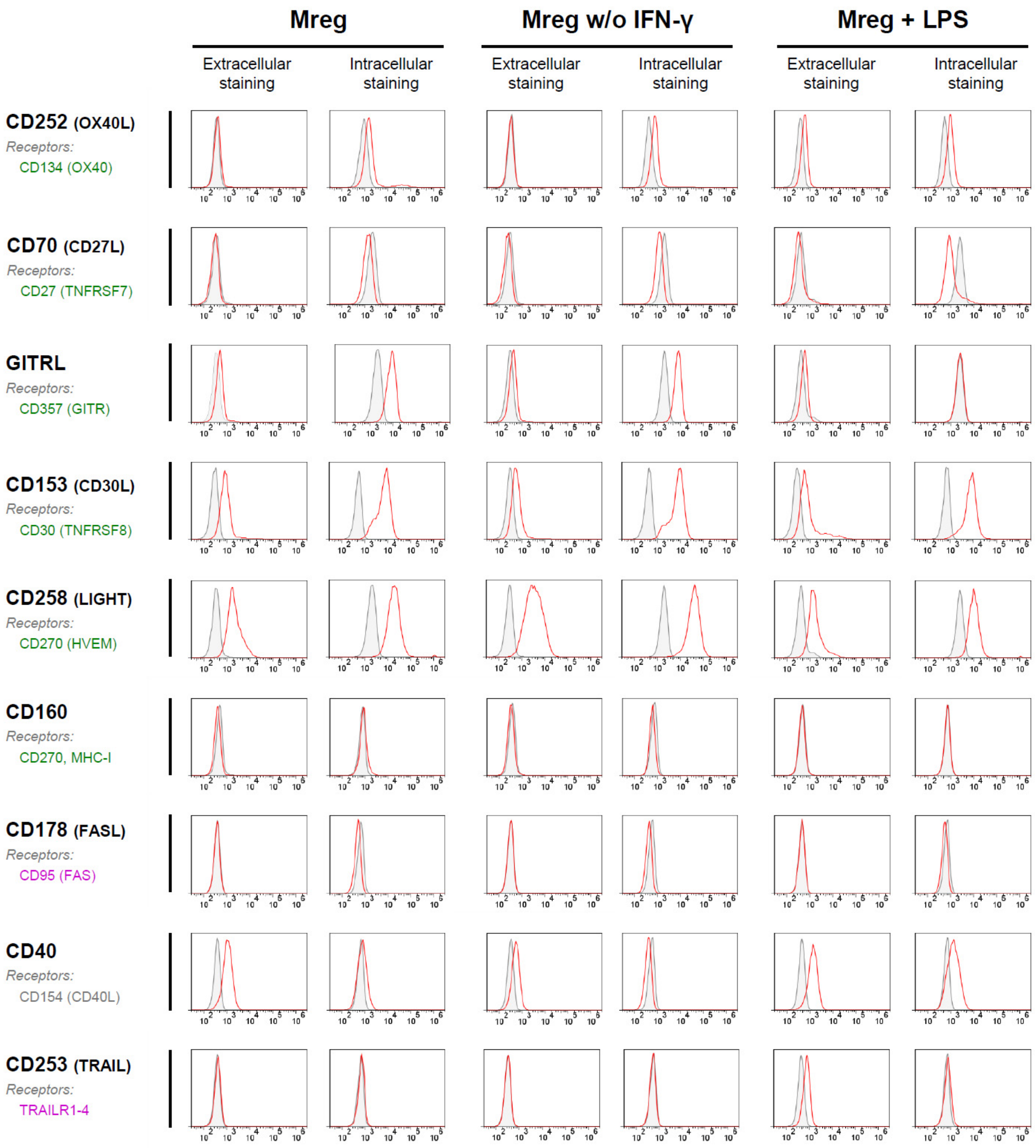

Expression of co-stimulatory and apoptosis-inducing TNFRSF receptor ligands by Mregs. Intact (extracellular) or permeabilised (intracellular) human Mregs were stained for ligands of co-stimulatory (green) and apoptosis-inducing (purple) receptors. Comparing Mregs to Mregs without IFN-γ-stimulation (Mreg w/o IFN-γ) reveals the contribution of IFN-γ stimulation to the Mreg phenotype. Comparing Mregs to Mregs stimulated for 24-hours with 100 ng/ml LPS demonstrates the stability of ligand expression by human Mregs (representative of n=6).

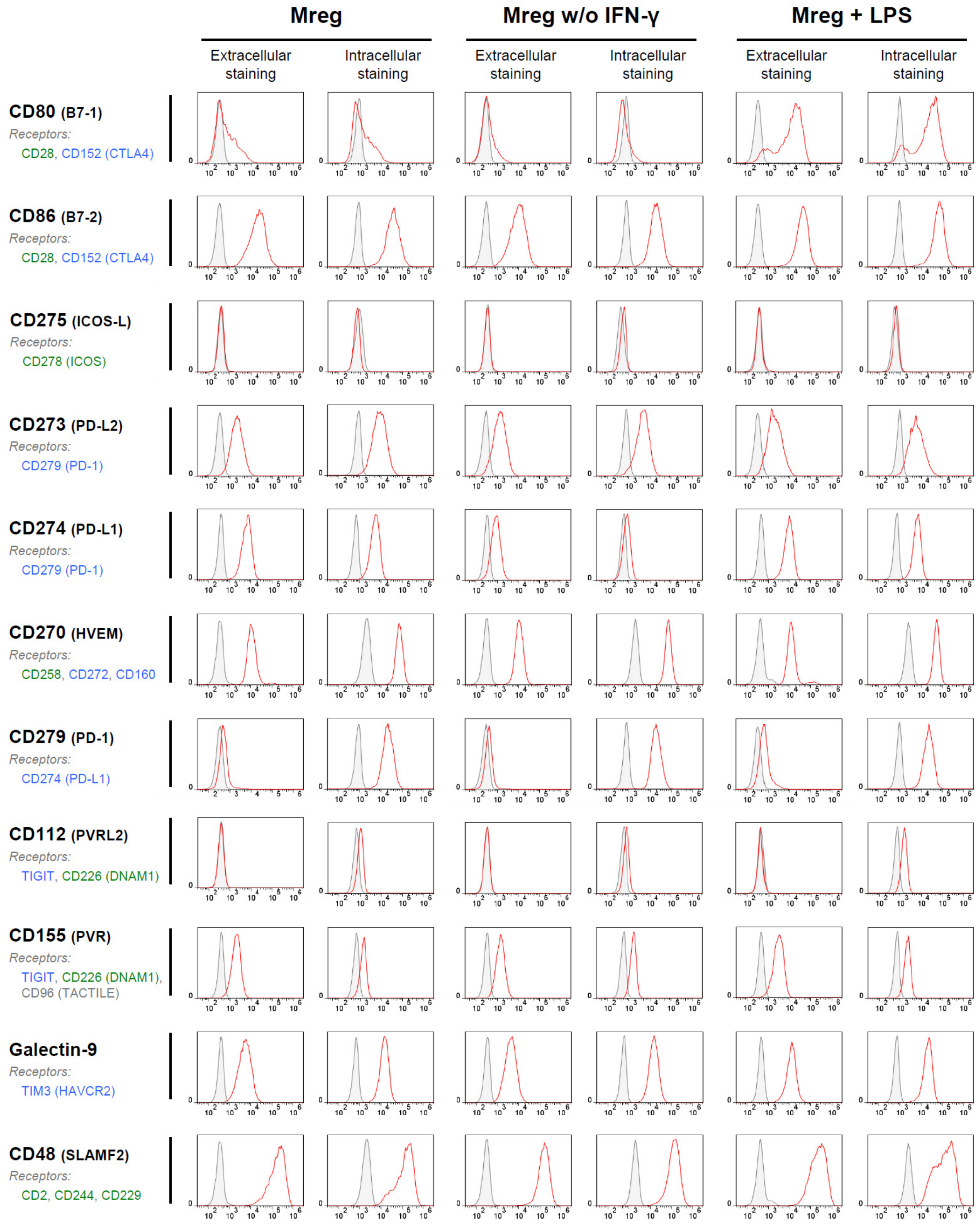

Expression of co-stimulatory and co-inhibitory IgSF receptor ligands by Mregs. Intact (extracellular) or permeabilised (intracellular) human Mregs were stained for ligands of co-stimulatory (green) and co-inhibitory (blue) receptors expressed by T cells. Comparing Mregs to Mregs without IFN-γ-stimulation (Mreg w/o IFN-γ) reveals the contribution of IFN-γ stimulation to the Mreg phenotype. Comparing Mregs to Mregs stimulated for 24-hours with 100 ng/ml LPS demonstrates the stability of ligand expression by Mregs (representative of n=6).

SUPPLEMENTARY FIGURE 2

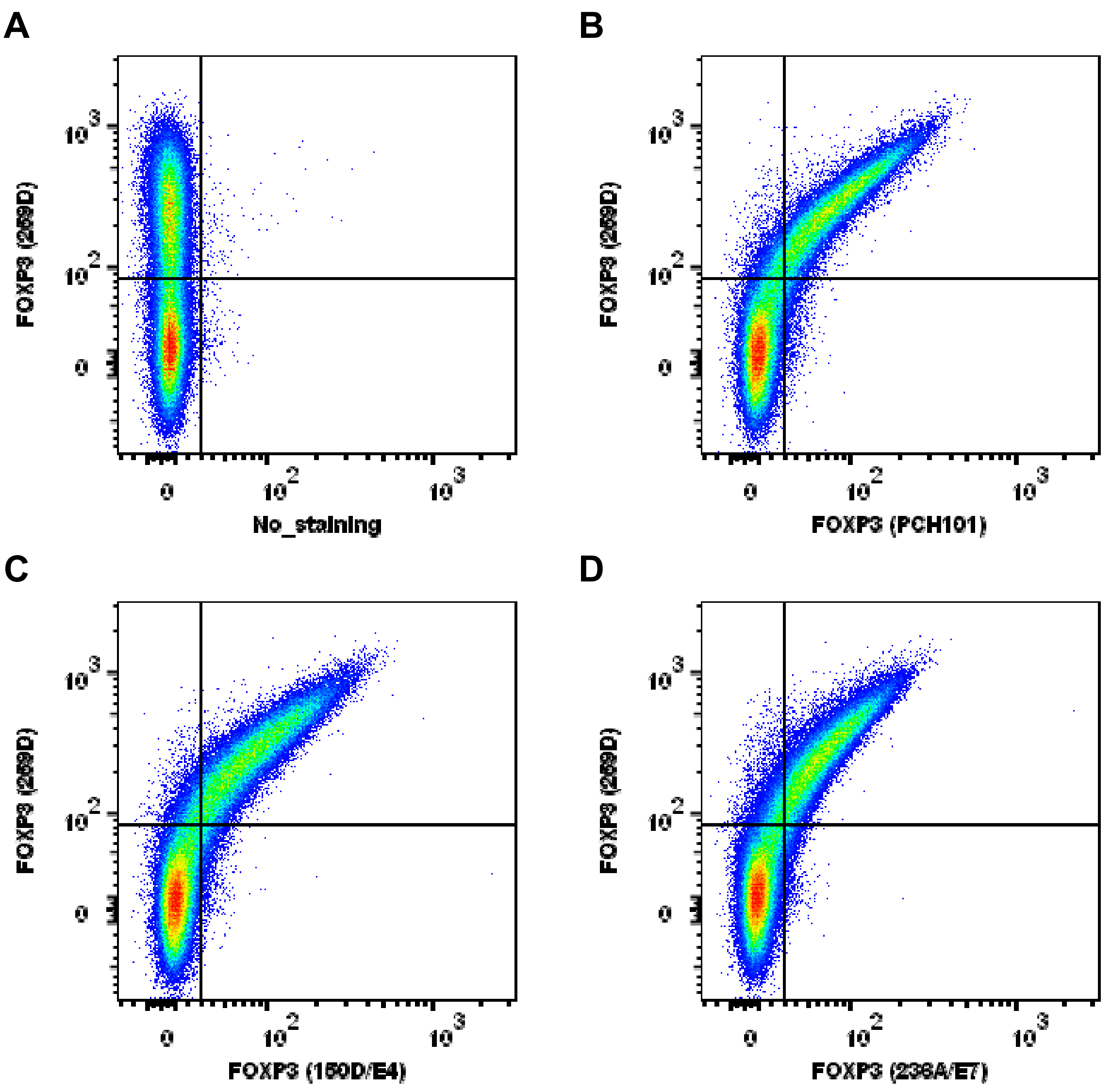

FoxP3 expression in Mreg-induced Tregs was detectable using four mAb that recognize different epitopes of FoxP3. **(A)** Naive human CD4+ T cells were cocultured with allogeneic Mregs prior to intracellular staining of FoxP3 with mAb clone 259D. Staining for FoxP3 using mAb 259D correlated with staining for FoxP3 using mAbs **(B)** PCH101, **(C)** 150D/E4 and **(D)** 236A/E7. mAb 150D/E4 recognises an epitope encoded by exon 2 of human FoxP3, which can be excised through alternative splicing of FoxP3 mRNA. Epitopes recognised by mAbs PCH101 and 236A/E7 are not affected by alternative splicing (representative of n=6).

SUPPLEMENTARY FIGURE 3

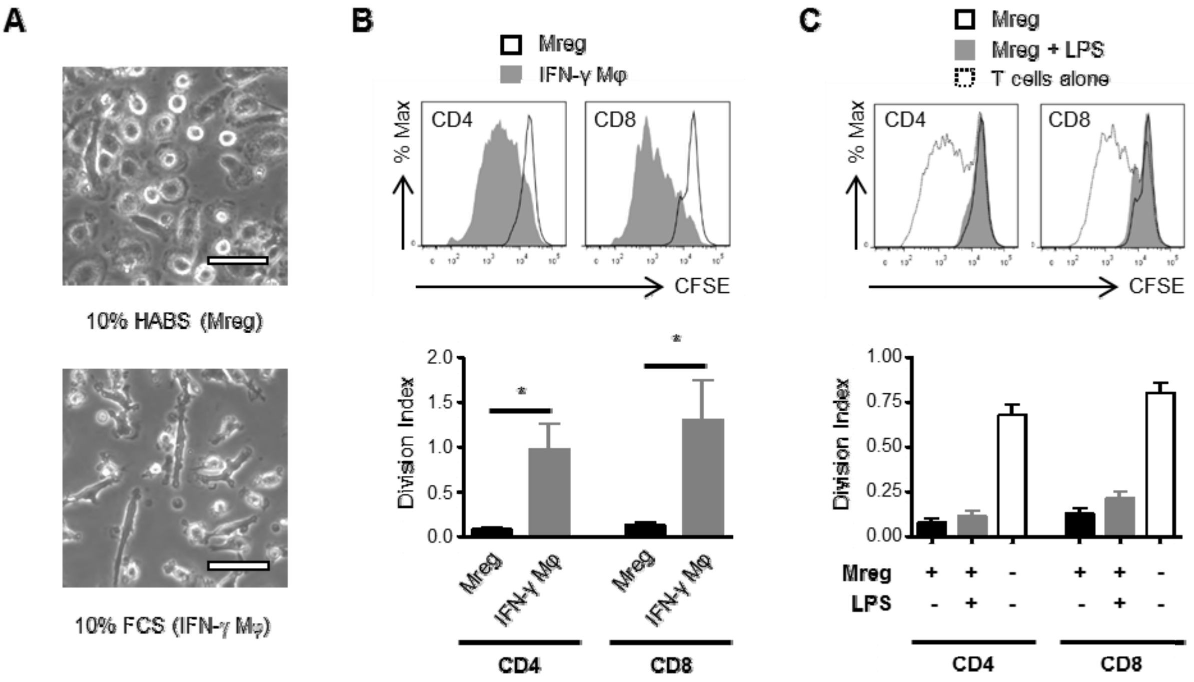

Justification of IFN-γ-Mφ as a relevant control cell. Human IFN-γ-stimulated macrophages (IFN-γ Mφ) are M1-polarised macrophages that arise from peripheral blood $CD14^{+}$ monocytes when cultured in medium containing fetal calf serum (FCS) and rhM-CSF. Human IFN-γ Mφ can be readily distinguished from Mregs by their morphology, $CD14^{high}$ $CD16^{high}$ phenotype and poor suppressor activity. **(A)** Human Mregs generated in medium supplemented with 10% human AB serum exhibit a characteristic spreading morphology, whereas IFN-γ Mφ grown in medium containing 10% FCS, but under otherwise identical conditions, develop into irregular elongated forms (bar = 50 μm). **(B)** In direct 1:1 co-cultures, Mregs strongly suppress PHA-stimulated $CD4^{+}$ and $CD8^{+}$ T cell proliferation, whereas equal numbers of IFN-γ Mφ do not (n=5; $CD4^{+}$: p=0.036; $CD8^{+}$: p=0.036; MW). **(C)** Mreg-mediated suppression of T cell proliferation was not disturbed by addition of 100 ng/ml LPS to the co-cultures (n=5; $CD4^{+}$: ns; $CD8^{+}$: ns; MW).

SUPPLEMENTARY FIGURE 4

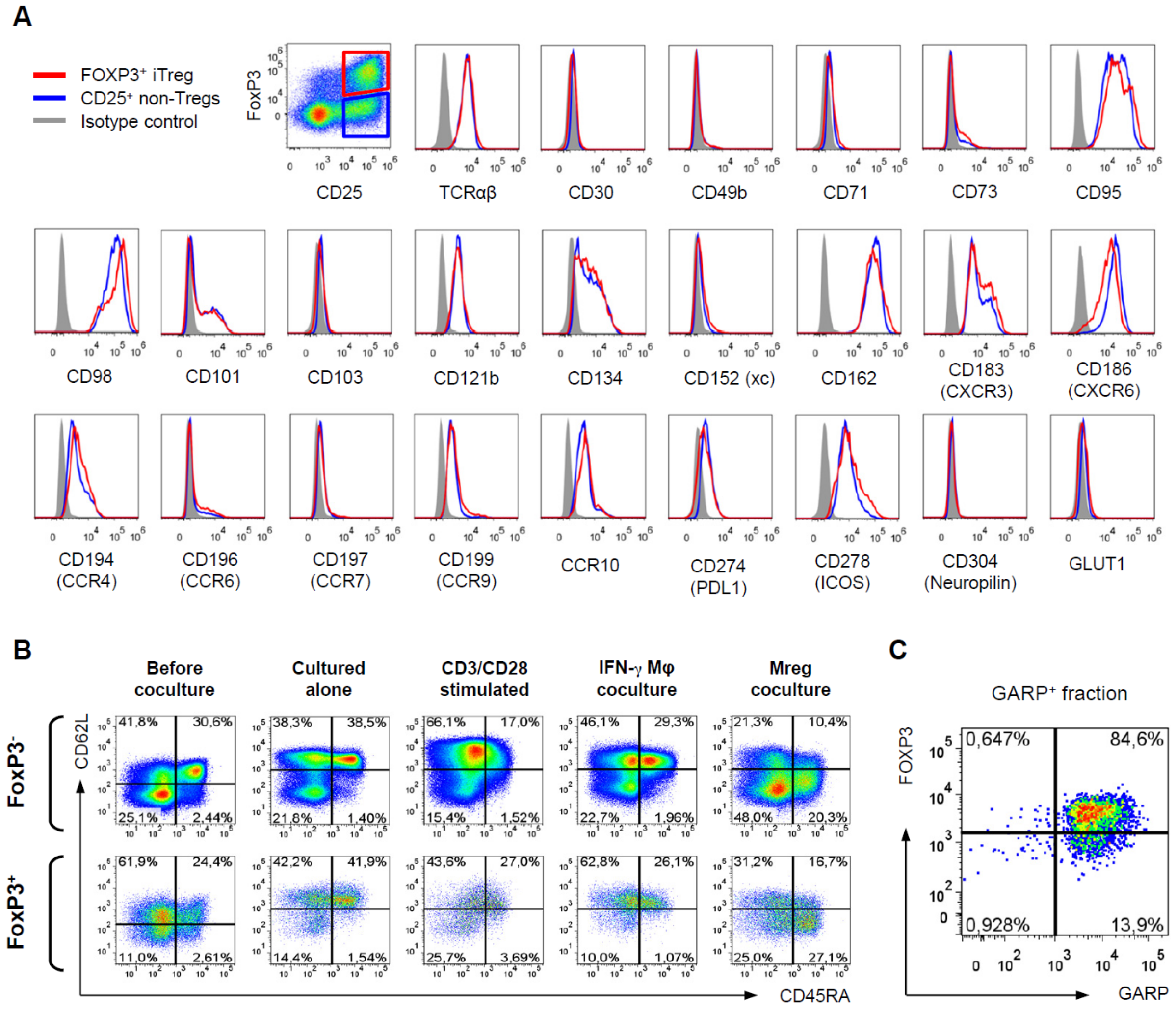

Comparative phenotyping of miTregs. Marker screening experiments were performed in order to establish whether FoxP3+ and FoxP3- T cells arising through allogeneic Mreg co-culture represented truly distinct populations. **(A)** A survey of markers expressed by FoxP3+ iTregs and CD25+ non-Tregs after allogeneic Mreg coculture (data representative of n=6 independent donor pairs). **(B)** Mreg coculture led to enrichment of CD62L- FoxP3- CD4+ non-Tregs, which were predominantly CD45RA-. By contrast, miTregs were mainly CD45RA+ (data represent means of n=6 independent donor pairs). **(C)** Representative plot showing GARP+ T cells isolated from Mreg-cocultures by flow-sorting. Using a GARP signal-amplification method, miTreg purities of 69.6 ± 14.2 % (n=3) were achieved.

SUPPLEMENTARY FIGURE 5

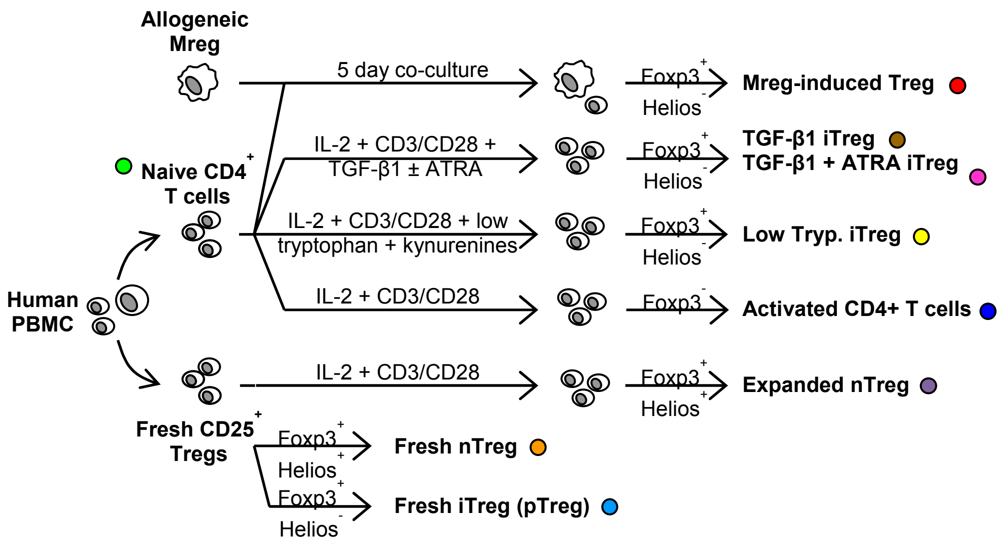

Phenotypic comparison of miTregs to other *in vitro*-derived iTregs, nTregs, pTregs and non-Tregs. To better understand the relationship between miTreg and other *in vitro*-derived iTregs, a phenotypic comparison was made between nine CD4+ T cell subtypes from n=6 donors considering 29 markers measured by flow cytometry. Colour coding corresponds to that shown in Figure 3F. Three alternative preparations of *in vitro*-derived human iTregs were differentiated from negatively-isolated human CD4+ naïve T cells. "Low tryptophan iTregs" were generated over 5 days in tryptophan-free medium supplemented with 10% dialyzed heat-inactivated human AB serum, antibiotics (penicillin-streptomycin), 4 µM tryptophan and 300 IU IL-2. Cultures were stimulated with CD3/CD28 MACSi-beads and 10 µM each of L-kyneurinine, quinolic acid/2,3 pyridinedicarboxilyc acid, anthranilic acid, 3-Hydroxy-anthranilic acid and 3-Hydroxy-DL-kyneurinine. "TGFβ-induced iTregs" were generated in X-Vivo 10 medium supplemented with GlutaMax, 300 IU IL-2 and 5 ng/ml TGF-β_1. Cultures were stimulated with CD3/CD28 MACSi-beads. "ATRA + TGFβ-induced iTregs" were cultured as TGFβ-induced iTregs except that medium was also supplemented with 10 nM all-*trans* retinoic acid (ATRA). "Expanded nTreg" were generated from bead-sorted CD4+ CD25+ nTregs cultured in X-Vivo 10 medium supplemented with GlutaMax and 300 IU IL-2. Cultures were stimulated with CD3/CD28 MACSi-beads. "Activated CD4+ T cells" were generated from bead-sorted naïve CD4+ T cells cultured in X-Vivo 10 medium supplemented with GlutaMax and 300 IU IL-2. Cultures were stimulated with CD3/CD28 MACSi-beads.

SUPPLEMENTARY FIGURE 6

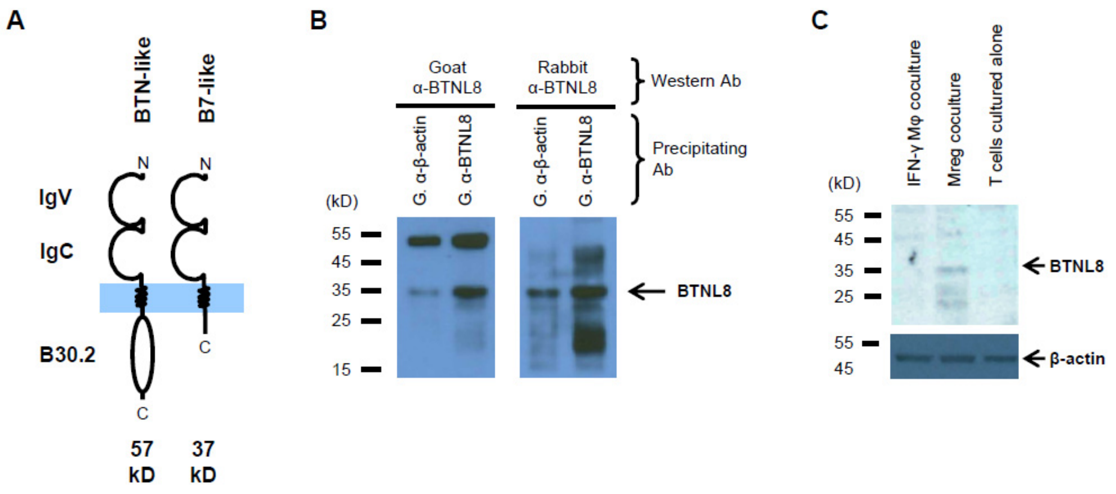

Detecting BTNL8 protein expression using commercial polyclonal antibodies. (A) Six putative alternatively-spliced isoforms of BTNL8 have been reported, ranging in predicted molecular weight from 37 kD to 57 kD (Uniprot Q6UX41). According to their structural likenesses, the two predominant isoforms have been categorized as B7-like or BTN-like. The BTN-like variant comprises extracellular IgV and IgC domains, a transmembrane region and an intracellular B30.2 domain. The B7-like variant shares the same extracellular IgV and IgC domains, but due to alternative splicing resulting in a frameshift, it has a different transmembrane region and only a short intracellular C-terminal domain. **(B)** A selection of polyclonal antibodies (pAbs) raised against the extracellular portion of full-length BTNL8 is commercially available; however, none of these reagents is well-characterised. The specificities of two pAbs were assessed by IP-Western. BTNL8 was precipitated from lysates of Mreg cocultured T cells using a goat pAb (sc-245053). As a negative control, a goat pAb (sc-1616) was used to precipitate β-actin. BTNL8 protein was then detected by Western blotting using either the goat α-BTNL8 pAb or a rabbit α-BTNL8 pAb (PA5-24933). In agreement with the PCR data, both anti-BTNL8 pAbs detected a precipitated protein at ~37-kD, but no protein at 57-kD. Therefore, we can be confident that Mreg-cocultured T cells express the B7-like isoform of BTNL8, but not the BTN-like variant, and that both the goat and rabbit pAbs are reactive with this isoform. **(C)** Western blot for BTNL8 using a goat α-BTNL8 pAb from T cells cocultured with allogeneic Mregs or IFN-γ-Mφ. A band corresponding to BTNL8 was detected at 37 kD.

SUPPLEMENTARY FIGURE 7

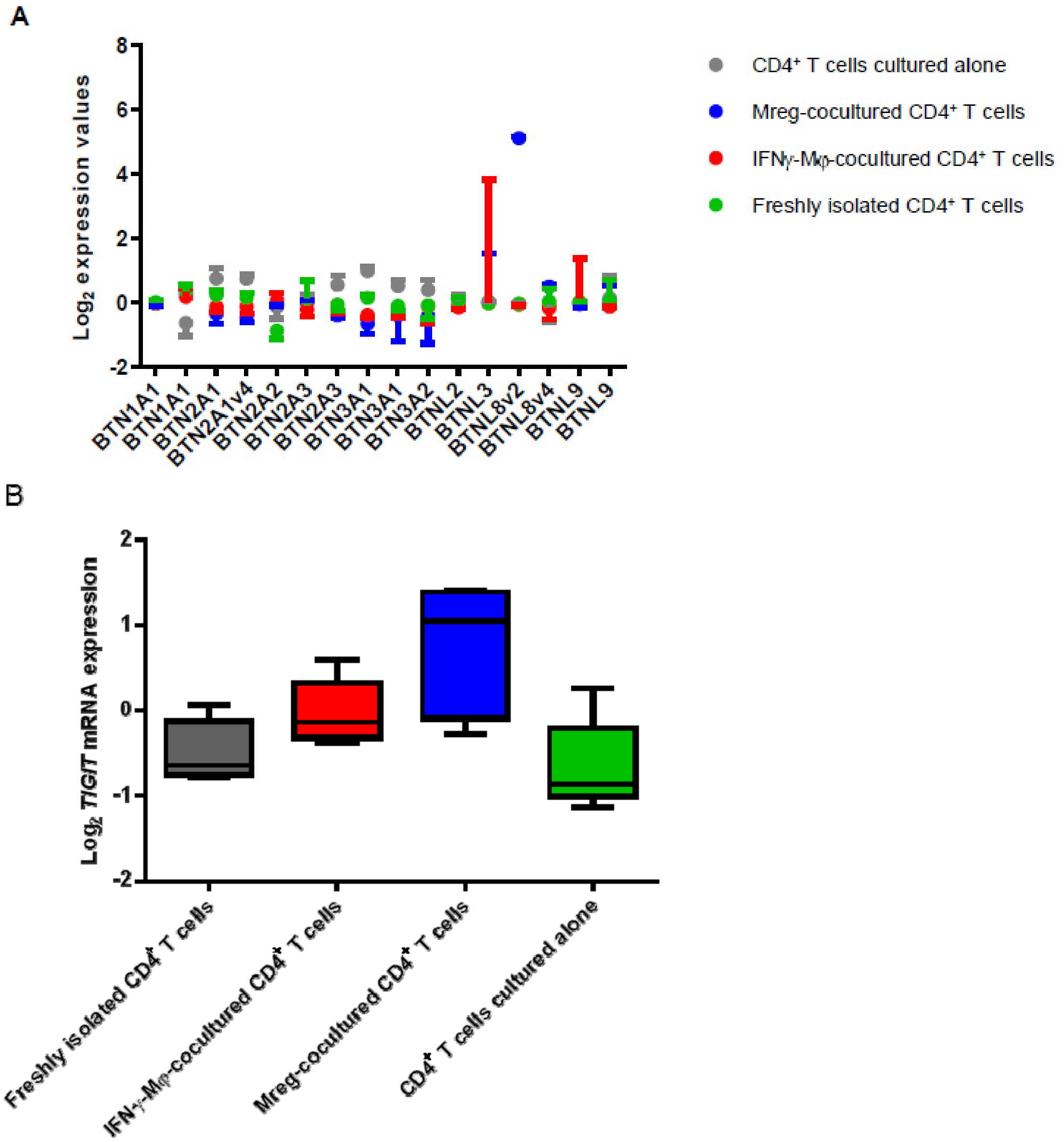

Screening for coinhibitory receptor expression as markers of Mreg-induced Tregs. (A) Log_2-transformed median-centered mRNA expression values for BTN and BTNL family members. Values are median ± IQR (n=5). **(B)** Screening for expression of IgSF coinhibitory receptors identified TIGIT (alias WUCAM, VSIG9 or VSTM3) as a possible marker of Mreg-cocultured T cells (n=5).

SUPPLEMENTARY FIGURE 8

Uncropped gels from Figure 7D

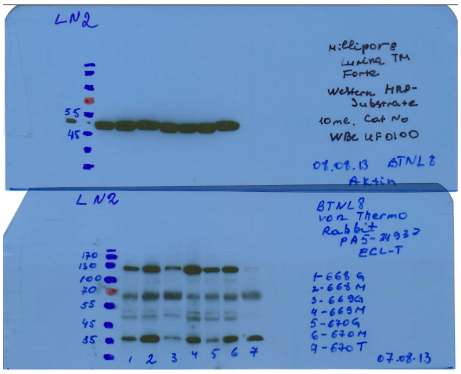

Uncropped gel from Figure 7J

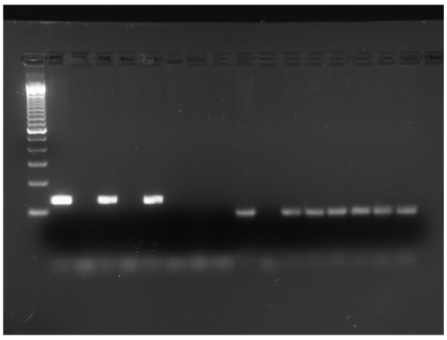

Uncropped gels from Supplementary Figure 6B

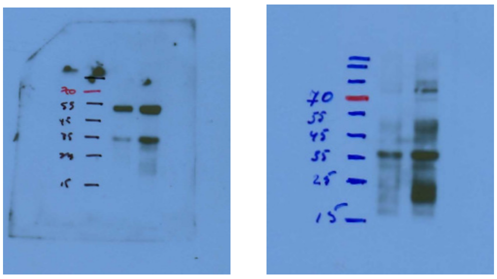

Uncropped gel from Supplementary Figure 6C

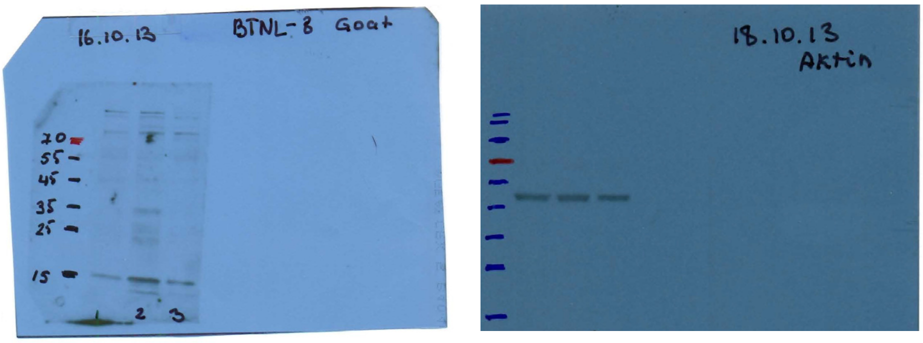

SUPPLEMENTARY TABLE 1

Pharmacological inhibitors added to co-cultures

Substance	Supplier	Cat. #	Preparation of stock solution
BTNL8-Fc	R&D	9359-BT	200 µg/ml in X-Vivo 10 medium
CTLA4-Fc	R&D	7268-CT	200 µg/ml in X-Vivo 10 medium
recombinant human Fc (rhFc)	R&D	110-HG	200 µg/ml in X-Vivo 10 medium
anti-BTNL8 mAb	R&D	MAB9359	200 µg/ml in X-Vivo 10 medium
Rabbit IgG mAb control	R&D	MAB1050	200 µg/ml in X-Vivo 10 medium
Normal goat IgG control	R&D	AB-108-C	200 µg/ml in X-Vivo 10 medium
mouse IgG1 control	R&D	MAB002	200 µg/ml in X-Vivo 10 medium
anti-IL-10 pAb	R&D	AF217-NA	200 µg/ml in X-Vivo 10 medium
anti-IL-10Rα pAb	R&D	AF274	200 µg/ml in X-Vivo 10 medium
anti-IL-10Rβ pAb	R&D	AF874	200 µg/ml in X-Vivo 10 medium
anti-TGFβ1 pAb	R&D	AF246-NA	200 µg/ml in X-Vivo 10 medium
anti-TGFβRII pAb	R&D	AF241	200 µg/ml in X-Vivo 10 medium
anti-IL-2 pAb	R&D	AF202-NA	200 µg/ml in X-Vivo 10 medium
anti-CD25 pAb	R&D	AF223-NA	200 µg/ml in X-Vivo 10 medium
anti-PP14 pAb	R&D	AF842	200 µg/ml in X-Vivo 10 medium
anti-CD80 mAb	R&D	MAB140	200 µg/ml in X-Vivo 10 medium
anti-CD86 mAb	R&D	MAB141	200 µg/ml in X-Vivo 10 medium
Tacrolimus (FK506)	Tocris	3631	10 mM in DMSO
Rapamycin	Tocris	1292	10 mM in DMSO
Dexamethasone	Tocris	1126	100 mM in DMSO
Mycophenolic acid (MPA)	Tocris	1505	100 mM in DMSO
dibenzazepine (DBZ)	Tocris	4489	100 mM in DMSO
1-D-methyl-tryptophan	Sigma	452483	10 mM in 0.1 M NaOH, adjust to pH 7.5
1-L-methyl-tryptophan	Sigma	447439	10 mM in 0.1 M NaOH, adjust to pH 7.5
SB 525334	Tocris	3211	100 mM in DMSO
R 268712	Tocris	5288	100 mM in DMSO
AGN 193109	Tocris	5758	20 mM in DMSO
Trimethoxyflavone (TMF)	Tocris	3859	5 mM in DMSO

SUPPLEMENTARY TABLE 2

Antibodies for flow cytometry

Antigen	Clone	Isotype	Conjugate	Supplier	Cat.#	Volume / stain (µl)
CD1a	HI149	mIgG1	APC	BD	559775	20
CD1c	AD5-8E7	mIgG2a	APC	Miltenyi	130-098-005	10
CD3	SK3	mIgG1	PerCP-Cy5.5	BD	332771	20
CD4	RPA-T4	mIgG1	V450	BD	560345	5
CD6	2H46D3B	mIgG2a	FITC	BC	B16492	10
CD8	SK1	mIgG1	FITC	BD	245772	20
CD11b	ICRF44	mIgG1	APC	BD	555388	20
CD11c	B-ly6	mIgG1	APC	BD	555392	20
CD14	MφP9	mIGg2b	APC	BD	345787	5
CD16	3G8	mIgG1	APC	BC	B00845	10
CD25	M-A251	mIgG1	APC-H7	BD	560225	5
CD27	M-T271	mIgG1	APC	BD	558664	20
CD27	REA499	REA(S)	FITC	Miltenyi	130-108-251	10
CD28	L293	mIgG1	PerCP-Cy5.5	BD	337181	20
CD28	REA612	REA(S)	PE	Miltenyi	130-109-520	10
CD30	Ki2	mIgG1	FITC	Miltenyi	130-098-687	10
CD33	P67.7	mIgG1	APC	BD	345799	10
CD39	REA739	REA(S)	FITC	Miltenyi	130-110-792	2
CD45	2D1	mIgG1	V500-C	BD	655873	5
CD45RA	2H4	mIgG1	FITC	BC	6603904	8
CD45RA	REA562	REA(S)	FITC	Miltenyi	130-113-927	2
CD48	REA292	rhIgG1	APC	Miltenyi	130-106-517	10
CD49b	REA188	REA(S)	PE	Miltenyi	130-100-331	10
CD57	NK1	mIgM	FITC	BD	555619	20
CD62L	REA615	REA(S)	PE	Miltenyi	130-109-555	10
CD64	22	mIgG1	APC	BC	IM3601	10
CD70	REA292	rhIgG1	APC	Miltenyi	130-104-358	10
CD71	M-A712	mIgG2a	APC	BD	551374	20
CD71	REA902	REA(S)	FITC	Miltenyi	130-115070	2
CD73	REA804	REA(S)	PE	Miltenyi	130-112-060	2
CD80	MAB104	mIgG1	APC	BC	B30642	10
CD83	HB15e	mIgG1	PE	BD	556855	20
CD85D	42D1	rIgG2a	PE	eBioscience	12-5149-42	5
CD85J	GHI/75	mIgG2b	PE	BD	551053	20
CD85K	ZM4.1	mIgG1	PE	eBioscience	17-5139-42	5
CD86	2331(FUN1)	mIgG1	APC	BD	555660	20
CD95	REA738	REA(S)	PE	Miltenyi	130-113-069	2
CD98	REA387	REA(S)	PE	Miltenyi	130-105-708	10
CD101	REA954	REA(S)	PE	Miltenyi	130-115-902	2
CD103	Ber-ACT8	mIgG1	FITC	BD	333155	20
CD112	R2.525	mIgG1	PE	BD	551057	20
CD121b	REA744	REA(S)	PE	Miltenyi	130-111-457	2
CD122	27302	mIgG1	PE	R&D	FAB224P	10
CD122	REA167	REA(S)	FITC	Miltenyi	130-100252	10
CD123	AC145	mIgG2a	APC	Miltenyi	130-098-873	20
CD127	HIL-7R-M21	mIgG1	APC-H7	BD	560225	5
CD127	REA614	REA(S)	FITC	Miltenyi	130-113-979	2

Antibodies for flow cytometry, continued

Antigen	Clone	Isotype	Conjugate	Supplier	Cat.#	Volume / stain (µl)
CD134	L106	mIgG1	PE	BD	340420	20
CD134	REA621	REA(S)	FITC	Miltenyi	130-109-664	10
CD137	REA765	REA(S)	FITC	Miltenyi	130-110-902	2
CD141	AD5-14H12	mIgG1	APC	Miltenyi	130-090-514	10
CD152	BNI3	mIgG2a	PE	BD	555853	20
CD152	REA1003	REA(S)	PE	Miltenyi	130-116-930	2
CD153	116614	mIgG2b	APC	R&D	FAB1028A	10
CD154	REA238	REA(S)	PE	Miltenyi	130-109-550	10
CD155	2H7CD155	mIgG1	PE	eBioscience	12-1550-41	5
CD160	BY55	mIgM	PE	BC	IM3657	20
CD162	REA319	REA(S)	PE	Miltenyi	130-104-754	10
CD163	GHI/61.1	mIgG1	APC	Miltenyi	130-098-643	10
CD169	7-239	mIgG1	APC	Miltenyi	130-098-643	10
CD172a	REA144	REA(S)	APC	Miltenyi	130-099-785	10
CD178	NOK-1	mIgG1	PE	eBioscience	12-9919-42	5
CD204	REA460	REA(S)	APC	Miltenyi	130-107-062	10
CD206	19.2	mIgG1	APC	BD	550889	20
CD209	DCN46	mIgG2b	APC	BD	551545	20
CD223	REA351	REA(S)	APC	Miltenyi	130-105-453	10
CD226	DX11	mIgG1	BB515	BD	565152	5
CD252	ik-1	mIgG2	PE	BD	558164	5
CD253	RIK-2	mIgG1	PE	BD	550516	5
CD258	REA244	rhIgG1	APC	Miltenyi	130-103-660	10
CD270	REA247	rhIgG1	APC	Miltenyi	130-101-609	10
CD273	MIH18	mIgG1	APC	BD	557926	20
CD274	MIH1	mIgG1	FITC	BD	558065	20
CD274	MIH1	mIgG1	PE	BD	557924	20
CD275	2D3/B7-H2	mIgG2b	PE	BD	552502	20
CD278	ISA-3	mIgG1	APC	eBioscience	17-9948-42	5
CD278	REA192	REA(S)	FITC	Miltenyi	130-100-734	10
CD279	MIH4	mIgG1	APC	BD	558694	20
CD279	PD1.3.1.3	REA(S)	FITC	Miltenyi	130-104-892	10
CD301	744812	mIgG2b	AF488	R&D	FAB48881G	10
CD304	AD5-17F6	mIgG1	APC	Miltenyi	130-090-900	10
CD357	REA1001	REA(S)	PE	Miltenyi	130-116-960	2
CD357	eBioAITR	mIgG1	APC	eBioscience	17-5875-41	5
CCR4	REA279	REA(S)	PE	Miltenyi	130-103-882	2
CCR6	REA190	REA(S)	PE	Miltenyi	130-100-377	10
CCR7	3D12	rIgG2a	PE	BD	552176	20
CCR9	REA469	REA(S)	PE	Miltenyi	130-107-169	10
CCR10	REA326	REA(S)	PE	Miltenyi	130-104-868	10
CXCR3	REA232	REA(S)	PE	Miltenyi	130-101-380	10
CXCR6	REA458	REA(S)	PE	Miltenyi	130-107-730	10
CX3CR1	REA385	REA(S)	APC	Miltenyi	130-105-838	10

SUPPLEMENTARY TABLE 3

Pre-designed primers for RT-PCR from Qiagen

Gene	Assay Name	Cat. No
Human BTNL8	Hs_BTNL8_1_SG	QT00039242
Human BTNL8	Hs_BTNL8_2_SG	QT01670991
Human CD3	Hs_CD3E_1_SG	QT00001792
Human GAPDH	Hs_GAPDH_2_SG	QT01192646
Human RPL13A	Hs_RPL13A_2_SG	QT02321333
Mouse Tigit	Mm_Tigit_2_SG	QT02372314
Mouse Il10	Mm_Il10_1_SG	QT00106169
Mouse Gapdh	Mm_Gapdh_3_SG	QT01658692
Mouse Rpl13a	Mm_Rpl13a_1_SG	QT00267197
