# Supplementary material for: TIGIT+ iTregs elicited by human regulatory macrophages control T cell immunity
Source: Nat Commun. 2018 Jul 20;9:2858. doi: 10.1038/s41467-018-05167-8 (PMC6054648; doi:10.1038/s41467-018-05167-8)
Supplement: Supplementary file 2 — Description of Additional Supplementary Files [file 41467_2018_5167_MOESM2_ESM.pdf]

Description of Additional Supplementary Files

File Name: Supplementary Data 1
Description:
Suppl. Data 1A One-way ANOVA to identify reporters that were highly and significantly regulated in any two of the comparator T cell populations. The spreadsheet contains normalized, log2-transformed, median-centered expression values for all reporters and samples. Red shading indicates relative up-regulation; green shading indicates relative down-regulation. Values given in italics are 'flagged' as potentially spurious, low-intensity (detection p-value ≥ 0.01) signals. In order to identify genes which are differentially regulated between any two cell types, one-way ANOVA ($p<0.01$, Benjamini-Hochberg correction for multiple testing) was conducted using GeneSpring GX (Agilent Technologies Inc). Only reporters with at least three valid signal intensity values in at least one cell type were considered. In addition to the p-value cut-off of 0.01, alternative fold-change cut-off values (5x, 10x, 20x) are presented. Applying the "auto-filter" option to columns E to H of the Excel sheet allows reporters fulfilling the different selection criteria to be selected.
Suppl. Data 1B Identification of differentially expressed genes between Mreg-cocultured CD4+ T cells and IFN-γ-Mϕ-cocultured CD4+ T cells. The spreadsheet is structured as follows: Columns A-C, AF & AG – annotation of the respective reporters (GeneSympbol, RefSeq, GeneID, descriptions and Gene Ontology annotations); columns D-G – sample medians; columns H&I – fold change in median expression between Mreg-cocultured CD4+ T cells and IFN-γ-Mϕ-cocultured CD4+ T cells; column J – uncorrected t-test p-value (two-tailed, unpaired, equal variance); column K – t-test p-value after Benjamini-Hochberg correction for multiple testing; columns L-Z – single normalized expression values as reporter-wise median-centered log2 ratios. Red shading indicates relative up-regulation; green shading indicates relative down-regulation. Values given in italics are 'flagged' as potentially spurious, low-intensity (detection p-value ≥ 0.01) signals.

File Name: Supplementary Data 2
Description: Functional annotation of reporters up-regulated in Mreg-cocultured CD4+ T cells compared to IFN-γ Mϕ-cocultured CD4+ T cells according to Gene Ontology (GO) pathways.
